# Long-term maintenance of human endometrial epithelial stem cells and their therapeutic effects on intrauterine adhesion

**DOI:** 10.1186/s13578-022-00905-4

**Published:** 2022-10-18

**Authors:** Wen He, Xuejing Zhu, Aijie Xin, Hongdan Zhang, Yiming Sun, Hua Xu, He Li, Tianying Yang, Dan Zhou, Hexin Yan, Xiaoxi Sun

**Affiliations:** 1 Obstetrics and Gynecology Hospital, Fudan University, Shanghai, China; 2 Shanghai Celliver Biotechnology Co. Ltd, Shanghai, China; 3 NHC Key Lab of Reproduction Regulation (Shanghai Institute for Biomedical and Pharmaceutical Technologies), Fudan University, Shanghai, China; 4 Shanghai Ji Ai Genetics and IVF Institute, Obstetrics and Gynecology Hospital of Fudan University, Shanghai, China; 5 Shanghai Key Laboratory of Female Reproductive Endocrine Related Diseases, Shanghai, China

**Keywords:** Endometrial stem cell, SSEA-1, Endometrial regeneration, Intrauterine adhesion

## Abstract

**Background:**

The human endometrium is a highly regenerative tissue that is believed to have two main types of stem cells: endometrial mesenchymal/stromal stem cells (eMSCs) and endometrial epithelial stem cells (eESCs). So far, eMSCs have been extensively studied, whereas the studies of eESCs are constrained by the inability to culture and expand them in vitro. The aim of this study is to establish an efficient method for the production of eESCs from human endometrium for potential clinical application in intrauterine adhesion (IUA).

**Results:**

Here we developed a culture condition with a combination of some small molecules for in vitro culturing and expansion of human SSEA-1^+^ cells. The SSEA-1^+^ cells exhibited stem/progenitor cell activity in vitro, including clonogenicity and differentiation capacity into endometrial epithelial cell-like cells. In addition, the SSEA-1^+^ cells, embedded in extracellular matrix, swiftly self-organized into organoid structures with long-term expansion capacity and histological phenotype of the human endometrial epithelium. Specifically, we found that the SSEA-1^+^ cells showed stronger therapeutic potential than eMSCs for IUA in vitro. In a rat model of IUA, in situ injection of the SSEA-1^+^ cells-laden chitosan could efficiently reduce fibrosis and facilitate endometrial regeneration.

**Conclusions:**

Our work demonstrates an approach for isolation and expansion of human eESCs in vitro, and an appropriate marker, SSEA-1, to identify eESCs. Furthermore, the SSEA-1^+^ cells-laden chitosan might provide a novel cell-based approach for IUA treatment. These findings will advance the understanding of pathophysiology during endometrial restoration which may ultimately lead to more rational clinical practice.

**Graphical Abstract:**

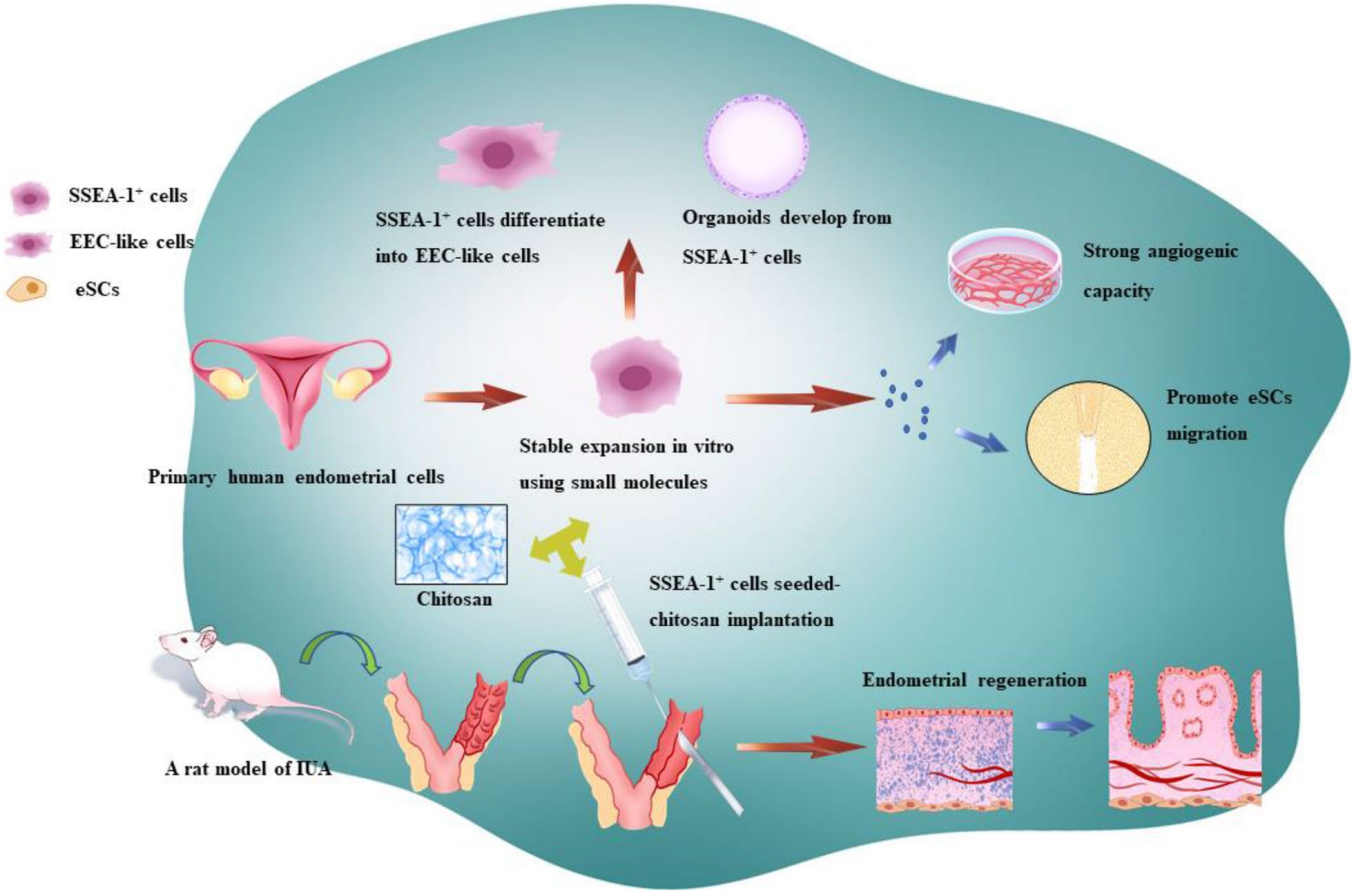

**Supplementary Information:**

The online version contains supplementary material available at 10.1186/s13578-022-00905-4.

## Introduction

The human endometrium is a unique tissue that exhibits robust regeneration which undergoes periodical transition of proliferation, differentiation and shedding during a woman’s reproductive life [1]. It has been demonstrated that the endometrial stem cells play an important role in supporting the tissue maintenance/regrowth [2]. The endometrium is structurally and functionally divided into basal layer and functional layer [3]. Previous studies demonstrated that the two main endometrial cell lineages, epithelium and stroma, of the functional layer may develop independently from their respective adult stem cells [4].

Endometrial mesenchymal stem cells (eMSCs), also known as endometrial mesenchymal stromal cells, were first identified as clonogenic stromal cells and shown to be capable of multi-lineage differentiation [5, 6]. With the increasing studies on eMSCs, the sushi domain containing-2 (SUSD2) was identified as a novel single marker for successfully purifying eMSCs [7]. By contrast, human endometrial epithelial stem cells (eESCs) are difficult to be cultured in vitro for long durations, with the in vivo phenotype not maintained using monolayer culture methods. According to recent reports, there are more stage-specific embryonic antigen-1 (SSEA-1) positive cells located in the epithelium of the proliferative phase than that of the secretory phase, and more in the basalis than in the functionalis epithelium [8, 9]. Furthermore, the SSEA-1^+^ cells exhibited progenitor activities in short-term monolayer in vitro culture and were presumed as eESCs [10]. However, these possibilities remain to be confirmed. Considering the highly proliferative potential of the in vivo eESCs during endometrial regeneration, we sought to determine whether an appropriate medium mimicking the in vivo milieu could be established to long-term culture and expand eESCs in vitro. It could not only help improve our understanding of the endometrium repairing mechanisms but offer an unlimited cell source for the generation of endometrial epithelial cells, which have broad applications in clinical medicine such as stem cell-based treatments for intrauterine adhesion (IUA).

IUA is a critical public health concern affecting approximately 19% of women after miscarriages [11]. However, current treatments including hysteroscopic lysis of adhesions, application of artificial hormone therapy, and placement of intrauterine devices cannot restore the structure and function of the endometrium, which leads to a high recurrence rate of severe IUAs. Presently, biological materials combined with stem cell transplantation strategies showed great prospects for endometrial regeneration [12]. Current research has found that transplantation with bone marrow mesenchymal stem cells, eMSCs and menstrual blood–derived stem cells into the uterine cavity through biological materials stimulate the growth of the endometrium [13]. It has become evident that eESCs in both the functional and basal layers are crucial for endometrial regeneration [14]. So far, the study on eESCs and IUA is very limited. Pioneering work over the past years suggested that a combination of growth factors and small molecules allowed for the stable culturing of adult progenitor cells, all of which exert their effect through signal regulatory pathways [15]. Following this rapidly advancing field, several small molecules, including CHIR99021, Y27632, PD0325901 and so on, were identified to shift a somatic cell toward alternative somatic identities or pluripotency in vitro [16, 17]. These prior findings suggested that such small-molecule compounds may allow for the stable culturing of human eESCs. Here, we developed a novel approach for isolation and expansion of human endometrial epithelial progenitor-like cells in vitro. Furthermore, we compared adult stem cell properties including self-renewal, proliferative potential, differentiation ability, organoid-forming capability, transcriptome expression and therapeutic effect on IUA rat models between the SSEA-1^+^ eESCs and SUSD2^+^ eMSCs.

## Results

### Human endometrial SSEA-1^+^ and SUSD2^+^ cells isolation and purification

**Fig. 1:**
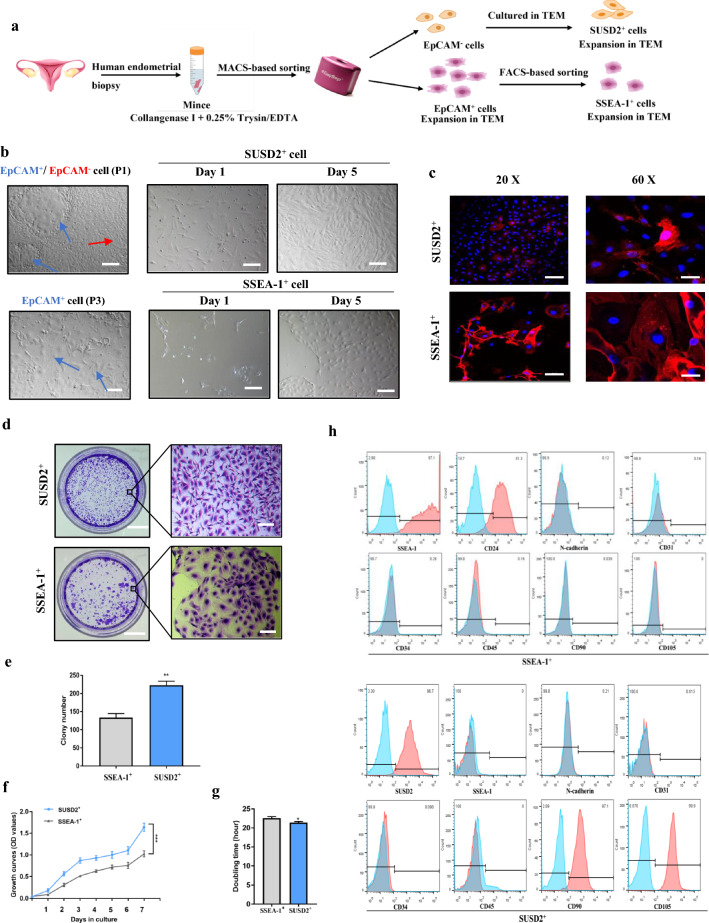
Generation of human SSEA-1^+^ cells in vitro. **a** Overview of the protocol used to obtain SSEA-1^+^ and SUSD2^+^ cells. **b** Light microscopy images of primary endometrial cells, P3 SUSD2^+^ and SSEA-1^+^ cells cultured in TEM at day 1 and day 5. Blue arrow, EpCAM^+^ cells; red arrow, EpCAM^−^ cells. Scale bars, 100 µm. **c** Immunofluorescence analyses demonstrating the expression of SUSD2 and SSEA-1. Scale bars, 50 µm in 20X, 20 µm in 60X. **d** Light microscopy images show typical 6-well plates of clone formation of SUSD2^+^ and SSEA-1^+^ cells by crystal violet staining. Scale bars, 1 cm in 1X, 100 µm in 10X. **e** SUSD2^+^ cells showed higher colony-forming unit numbers than SSEA-1^+^ cells (Error bars represent s.d.; n = 3 technical replicates; two-tailed unpaired t-test, **P < 0.01). **f** CCK-8 analyses demonstrating higher cell proliferation of SUSD2^+^ cells. (Error bars represent s.d.; n = 3 technical replicates from one donor; two-way ANOVA, *P* < 0.001). **g** Doubling time calculated for SUSD2^+^ and SSEA-1^+^ cells. Error bars represent s.d.; n = 3 technical replicates, *P < 0.05. **h** Flow cytometric analysis showing the proportion of positive-cells in the populations. Red, positive-cells; blue, negative controls

Based on previous findings regarding the survival and growth requirements for adult and mouse mammary gland progenitor cells in vitro, the candidates of the transition and expansion medium (TEM) were selected from a pool of growth factors and bioactive small molecules [17, 18]. All of the selected molecules are identified to date that provide enhancements of somatic cell reprogramming [19]. This led us to ask whether certain combinations of these molecules were an optimum culture medium for the survival and growth of SSEA-1^+^ cells. The whole experimental procedure for SSEA-1^+^ and SUSD2^+^ cells isolation and purification were schematically shown in Fig. 1a. To test this idea, we firstly isolated human primary EpCAM^+^ cells from human endometrial tissues using MACS-based sorting to exclude EpCAM^−^ stromal cells (Additional file 1: Figure S1a). Contrary to the general notion that endometrial EpCAM^+^ cells do not proliferate in vitro, we observed the proliferation of endometrial EpCAM^+^ cells in the presence of multiple combinations of these factors (Fig. 1b) [20]. The results shown in Additional file 1: Figure S4b indicate that the EpCAM^+^ cells expressed EpCAM, Cytokeratin and E-cadherin while Vimentin was only observed in EpCAM^−^ cells.

SSEA-1 is a cell surface glycan which indicates an undifferentiated state of the committed endometrial progenitors [8, 10]. With increase in number of passages, the SSEA-1 expression of the sorted EpCAM^+^ cells was gradually elevated when cultured in TEM (Additional file 1: Figure S1b). At the 3^rd^ passage, we used FACS-based sorting to achieve purified SSEA-1^+^ cells. Next, we optimized the culture condition for maintenance and expansion of the SSEA-1^+^ cells. Upon the sequential withdrawal of individual factors from TEM, the proliferative capacity decreased accordingly, which suggested that each factor had a crucial influence on the growth of SSEA-1^+^ cells (Additional file 1: Figure S2a,b). Notably, proliferating cells were scarcely observed without Y2732 or CHIR99021 treatment, which were considered to be key factors for stable culturing of adult mammary progenitor cells (Additional file 1: Figure S2a) [16, 21, 22]. Consistent with this pattern, lower numbers of colonies were shown in cells cultured with additional Nicotamide or PD0325901 (Additional file 1: Figure S2a,b). These results suggest that the synergistic relationship among these factors in TEM has a strong effect on stably expanding SSEA-1^+^ cells. Supporting this suggestion, similar proliferative capacity was found in three donor-derived SSEA-1^+^ cells cultured in TEM (Additional file 1: Figure S2c).

The purified SSEA-1^+^ cells were positive for SSEA-1 both by immunofluorescence staining and flow cytometry (Fig. 1c, h). Flow cytometry analysis revealed that the SSEA-1^+^ cells purity was 97%, accompanied by a strong expression of CD24 (81.3%) and they were negative for N-cadherin, CD31, CD34, CD45, CD90, and CD105 (Fig. 1h). Typical 3D confocal laser scanning image of SSEA-1^+^ and SUSD2^+^ cells were presented in supplemental material. The SSEA-1^+^ cells exhibited a homogeneous whorled or polyhedral morphology and formed tight complex clone structures **(**Fig. 1b). The immunohistochemistry of the proliferative endometrial tissues showed that all endometrial luminal and glandular epithelial cells were positive for Cytokeratin. However, only a small proportion of endometrial epithelial cells were positive for SSEA-1, whereas all epithelial cells were negative for N-cadherin **(**Additional file 1: Figure S1c).

According to Masuda et al., the SUSD2 was identified as a novel single marker for purifying human eMSCs [7]. After expansion in TEM, almost all P3 endometrial EpCAM^−^ cells were positive for SUSD2 both by flow cytometry and immunofluorescence staining (Fig. 1c, h). To confirm the eMSCs phenotype, FACS analyses revealed that the SUSD2^+^ cells were positive for CD 90 and CD105, and negative for SSEA-1, N-cadherin, CD31, CD34 and CD45 (Fig. 1h). These results were consistent with previous reports about eMSCs’ surface markers [23]. The SUSD2^+^ cells presented a characteristic fibroblast-like morphology and were arranged in a spiral pattern (Fig. 1b). These results indicate that the SUSD2^+^ cells, or human eMSCs, were successfully isolated and identified in the present culture system.

Although both SSEA-1^+^ and SUSD2^+^ cells displayed great colony formation potential when cultured in TEM, the SUSD2^+^ cells had higher colony-forming unit numbers (223 ± 6) than SSEA-1^+^ cells (134 ± 6) (Fig. 1d, e). In addition, the cell proliferative capacity of SUSD2^+^ cells was significantly higher than SSEA-1^+^ cells (Fig. 1f), accompanied by a slightly lower mean population doubling time (21.4 ± 0.2 h vs. 22.6 ± 0.2 h, Fig. 1g). We then asked whether SSEA-1^+^ cells could undergo long-term culture without losing proliferative capacity. Previous studies demonstrated that OCT-4, NANOG, SOX2 and SOX9 are expressed in adult stem cells under a less-differentiated state [24–26]. We found that the late-passage SSEA-1^+^ cells displayed reduced proliferative capacity and decreased mRNA expressions of *OCT-4*, *NANOG*, *SOX2* and *SOX9* (Additional file 1: Figure S3a,c). The morphology of P10 SSEA-1^+^ cells became apical, and the colonies were found scattered with an increase in apoptosis (Additional file 1: Figure S3e, f). The apoptotic rate of P10 SSEA-1^+^ cells was much higher than that of P1 SSEA-1^+^ cells (71.3% vs. 5.6%). In contrast, the SUSD2^+^ cells showed no signs of senescence with passages (Additional file 1: Figure S3d). There were no significant differences of mRNA expressions of *OCT-4*, *NANOG*, *SOX2* and *SOX9* among the P1, P6 and P10 SUSD2^+^ cells (Additional file 1: Figure S3b). The karyotype analysis revealed that all the three independently established P10 SSEA-1^+^ and SUSD^+^ cells maintained normal diploid karyotypes (Additional file 1: Figure S3g).

### Gene expression profiles between SSEA-1^+^ and SUSD2^+^ cells

**Fig. 2:**
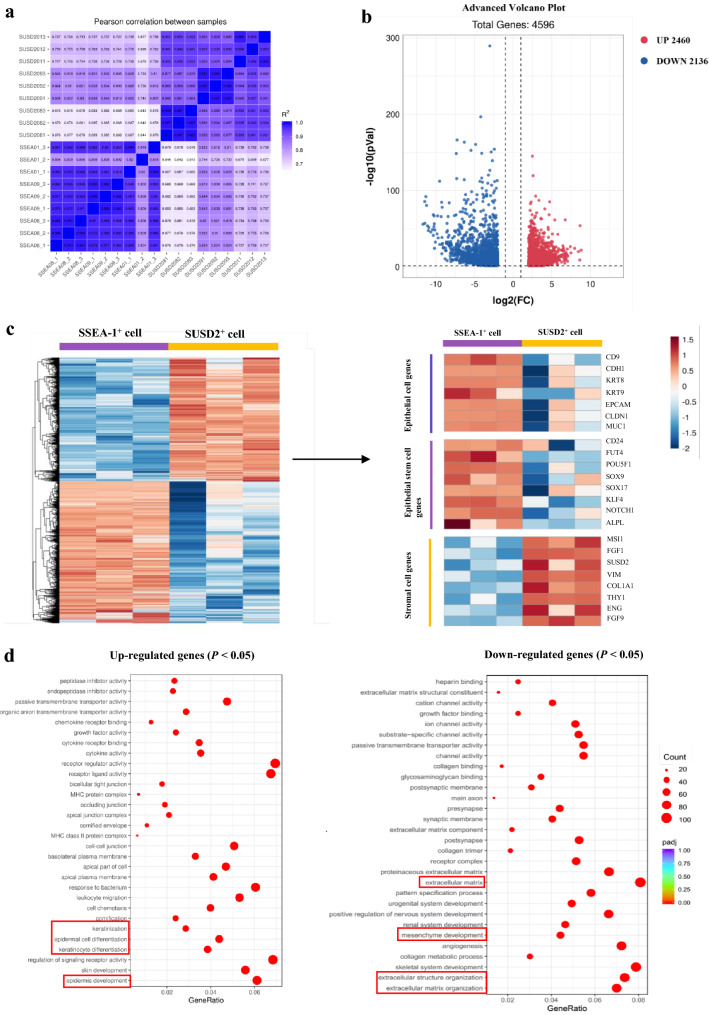
RNA sequence–based transcriptome profiles of SSEA-1^+^ and SUSD2^+^ cells. **a** Pairwise correlation heatmap of RNA-seq samples based on the Pearson correlation of log gene expression values for all genes. **b** Volcano plot analysis of the differentially expressed mRNA after comparison between SSEA-1^+^ and SUSD2^+^ cells (> twofold changes and *P* < 0.05). **c** Left: Heat-map of up- or down-regulated genes in SSEA-1^+^ cells compared with SUSD2^+^ cells. Right: Heatmap on the selected epithelial genes, endometrial stem cell-specific genes and stromal genes between SSEA-1^+^ and SUSD2^+^ cells (> twofold changes and *P* < 0.05). **d** GO analysis of differentially expressed mRNAs. Top 30 GO biological processes enriched among up-regulated and down-regulated genes in SSEA-1^+^ cells compared with SUSD2^+^ cells. The color key is shown on the right. The most highly enriched GO categories are indicated in red. The size of the circles reflects the frequency of the GO term

To further characterize the function of SSEA-1^+^ and SUSD2^+^ cells, RNA-seq analysis was performed to compare the gene expression levels in these two cell populations from three donors. Pearson correlation analysis revealed that the SSEA-1^+^ and SUSD2^+^ cells exhibited a high degree of phenotypic homogeneity (Fig. 2a). The volcano plot for hierarchical clustering showed that 2460 genes were up-regulated, and 2136 genes were down-regulated in SSEA-1^+^ cells among differentially expressed 4596 genes (twofold change, *P* < 0.05, Fig. 2b). Next, the global gene expression profiles from SSEA-1^+^ and SUSD2^+^ cells were subjected to unsupervised cluster analysis that identified discrete segregation between the two cells. Selected endometrial epithelial cell-specific genes were notably more highly expressed in SSEA-1^+^ cells, whereas SUSD2^+^ cells expressed relatively higher endometrial stromal cells-specific genes (Fig. 2c). In line with previous findings, the epithelial stem cell-specific genes were significantly more highly expressed in SSEA-1^+^ cells, which were related to stem cell maintenance [20]. Gene Ontology (GO) term analysis of the up-regulated genes in the SSEA-1^+^ cells showed great relationships with keratinocyte differentiation, epidermis development, keratinization, epidermal cell differentiation, etc. (Fig. 2d). Conversely, the down-regulated genes were related to mesenchyme development, extracellular matrix and extracellular matrix/structure organization (Fig. 2d). These gene signatures support the conjecture that the SSEA-1^+^ cells were associated more with endometrial epithelial development and differentiation than SUSD2^+^ cells.

### Efficient endometrial epithelial cell-like cells (EEC-like) differentiation of SSEA-1^+^ cells

**Fig. 3:**
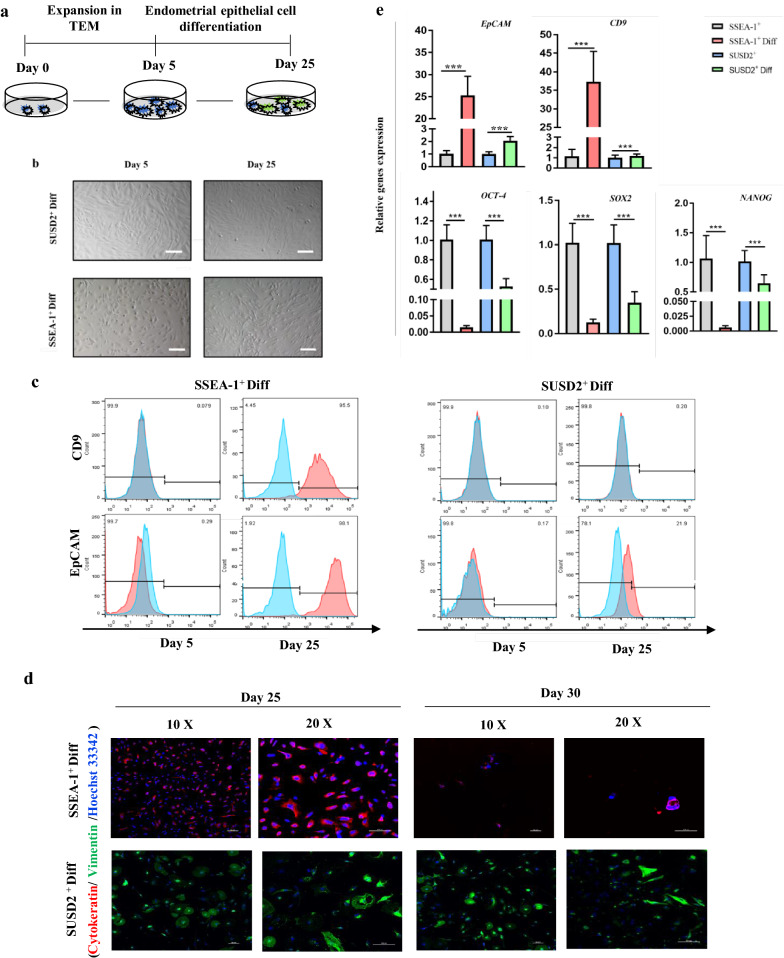
SSEA-1^+^ cells exhibit endometrial epithelial stem cell phenotype. **a** Overview of the protocol for SSEA-1^+^ and SUSD2^+^ cells differentiation into EEC-like cells. **b** Morphologic change of SSEA-1^+^ and SUSD2^+^ cells after differentiation. Scale bars, 100 µm. SSEA-1^+^/SUSD2^+^ Diff, SSEA-1^+^/SUSD2^+^ cells differentiation. **c** Flow cytometric analysis showing the proportion of EpCAM and CD9 positive-cells in SSEA-1^+^ or SUSD2^+^ cells at day 0 and day 20 of differentiation. Red, positive-cells; blue, negative controls. **d** Immunofluorescence analyses demonstrating the expression of Cytokeratin and Vimentin in EEC-like cells differentiated from SSEA-1^+^ and SUSD2.^+^ cells. **e** Gene expression levels for *EpCAM*, *CD9*, *OCT-4*, *SOX2* and *NANOG*. Expression normalized to *β-actin* (two-tailed unpaired t-test, Error bars represent s.d., n = 3; n.s., non-significant; *P < 0.05, **P < 0.01, ***P < 0.001)

In normal endometrium, estrogen (E2) stimulates the proliferation of endometrial epithelial cells. Previous studies reported that differentiation medium containing E2 could induce stem cells to differentiate into EEC-like cells [27–29]. This let us to hypothesize that the SSEA-1^+^ cells might differentiate into EEC-like cells with the E2-based differentiation medium. After incubation in differentiation medium for 20 days, the morphology of SSEA-1^+^ cells changed from a whorled clone to a fusiform shape and the clone structures were not well-knit anymore (Fig. 3b). With the time extension, apoptosis of SSEA-1^+^ cells increased greatly. By contrast, no obvious morphologic changes of SUSD2^+^ cells were found (Fig. 3b).

Differentiated EEC-like cells were also detected by flow cytometric analysis for EpCAM and CD9 expressions. The epithelial cell markers were remarkably up-regulated in the EEC-like cells differentiated from SSEA-1^+^ cells, with over 95% CD9 and over 98% EpCAM expressions (Fig. 3c). A low level of EpCAM (21.9%) expression was found in EEC-like cells differentiated from SUSD2^+^ cells, however, these cells were negative for CD9 expression (Fig. 3c). As shown in Fig. 3d, the immunofluorescence analysis revealed that the EEC-like cells differentiated from SSEA-1^+^ cells were positive for Cytokeratin and negative for Vimentin. On the contrary, the EEC-like cells differentiated from SUSD2^+^ cells were positive for Vimentin and negative for Cytokeratin. The qPCR analysis of EEC-like cells differentiated from SSEA-1^+^ cells demonstrated the obviously down-regulation of transcripts related to pluripotency (*OCT-4*, *NANOG* and *SOX2*), accompanied by the significantly up-regulation of transcripts of epithelial, lineage-associated transcripts (*CD9* and *EpCAM*) after day 20 differentiation, as compared with SSEA-1^+^ cells cultured in TEM (Fig. 3e). Nevertheless, the expressions of *CD9* and *EpCAM* increased slightly and the *OCT-4*, *NANOG* and *SOX2* decreased slightly after differentiation of SUSD2^+^ cells. As expected, SSEA-1^+^ cells differentiated into EEC-like cells with a high differentiation efficiency whereas SUSD2^+^ cells failed to do so. Although MSCs were reported with potential ability that they could cross lineage barriers to differentiate into endometrial epithelial cells [30, 31], SUSD2^+^ cells seemed to stay in a mesenchymal state with TEM, resulting in a low differentiation efficiency in the present differentiation system. At the late period of differentiation of SSEA-1^+^ cells, the EEC-like cells displayed less proliferation and increased cell apoptosis (Fig. 3d). This appearance is similar to the epithelial cells in monoculture [32], indicating that the SSEA-1^+^ cells transformed from a progenitor state to a maturation state after differentiation.

### Establishment of SSEA-1^+^ cells-derived organoid in 3D culture

**Fig. 4:**
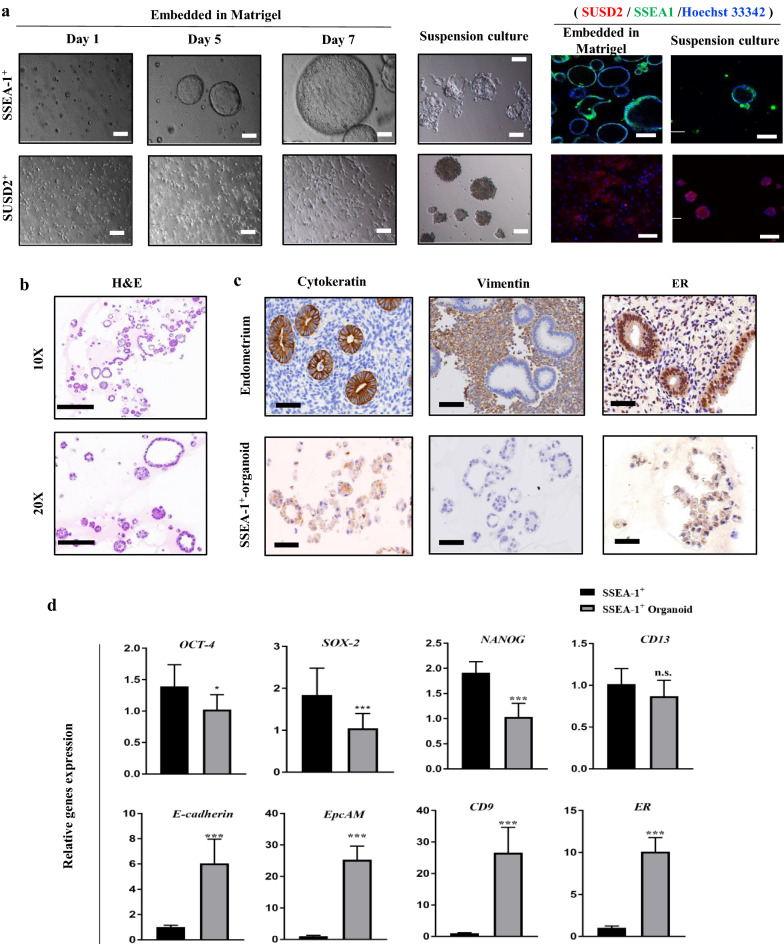
Organoids develop from SSEA-1^+^ cells and reproduce the phenotype of the epithelium. **a** Morphologic change and immunofluorescence analyses of SSEA-1^+^ and SUSD2^+^ cells embedded in Matrigel and in suspension culture. Scale bars, 50 µm in light microscopy images, 100 µm in confocal images. **b** H&E staining for organoids from SSEA-1^+^ cells. Scale bars, 200 µm in 10X, 100 µm in 20X. **c** The Cytokeratin, Vimentin and ER immunoexpression in the endometrium of proliferative phase and SSEA-1^+^-organoids. Scale bars, 100 µm in endometrium, 50 µm in SSEA-1^+^-organoids. **d** qPCR analyses for the expressions of *OCT-4*, *SOX2*, *NANOG*, *E-cadherin*, *CD13*, *EpCAM*, *CD9* and *ER* in SSEA-1^+^ cells and organoids from SSEA-1.^+^ cells. Expression normalized to *β-actin* (n = 3 donors; two-tailed unpaired t-test, n.s., non-significant, * *P* < 0.05, ***P < 0.001)

One common feature to all human organoid is that they are derived from pluripotent stem cells or adult stem cells [33]. Recent efforts to establish endometrial epithelial organoid have been successful with the freshly isolated endometrial cells [34–36], suggesting the presence of eESCs. Under the culture system utilizing Matrigel and TEM, the SSEA-1^+^ cells rapidly self-organized into spheroid-like or organoid-like structures with a hollow center within 7 days that further expanded in size after passages (Fig. 4a). In contrast, the SUSD2^+^ cells could not form any spheroids but the typical fibroblast-like appearance. While cultured in low-attachment wells, the SUSD2^+^ cells formed solid and non-opaque spheres with no loss of cell aggregation for up to 10 days, indicating that the SUSD2^+^ cells-spheres were just the cell aggregation rather than organoids. On the other hand, hollow-center organoids were gained from the SSEA-1^+^ cells in suspension culture. The spheroid diameter of SSEA-1^+^ cells in suspension culture was far smaller than that in Matrigel culture. The immunofluorescence staining indicated that no cross-contamination between SSEA-1^+^ and SUSD2^+^ cells (Fig. 4a).

Organoids could remodel the epithelial compartment of a tissue without the essential presence of mesenchymal compartment of the tissue [37]. The typical HE staining results of SSEA-1^+^ cells forming organoids were shown in Fig. 4b. The SSEA-1^+^ cells forming organoids reproduced endometrial epithelium phenotype after 14 days culturing in TEM. The organoids expressed Cytokeratin and ER but Vimentin, mimicking the in vivo epithelium phenotype of the proliferative phase of human endometrial glands (Fig. 4c). In line with this expression, we found that addition of E2, although not strictly required, could slightly promote the expansion of the organoids (data not shown). Moreover, the mature epithelial genes including *E-cadherin*, *EpCAM*, *CD9* and *ER* were up-regulated and the pluripotency-maintaining genes (*OCT-4*, *SOX2* and *NANOG*) were down-regulated in SSEA-1^+^ cells forming organoids. The mRNA expression of *CD13*, the marker of the stromal lineage, was not altered after self-organized into organoid (Fig. 4d). Taken together, we established the human endometrial organoids that mimic the epithelial characteristics of the tissue from a purified long-term cultured human endometrial cells, instead of endometrial fragments or other co-culture systems, thereby providing a promising in vitro model for studying human endometrial biology in greater depth.

Obtaining the adult stem cells-derived organoids required essential niche factors to support stem cell activity [38]. Previous studies have demonstrated that epithelial proliferation and stem cell self-renewal are dependent on EGF and WNT activity and the long-term maintenance of human organoids requires inhibition of the TGF-β pathway [39, 40]. Our results demonstrated that the cytokine cocktail in TEM could maintain the adult stem cell biological characteristics of SSEA-1^+^ cells in both 2D and 3D cultures.

### SSEA-1^+^ cells had higher angiogenic potential than SUSD2^+^ cells

**Fig. 5:**
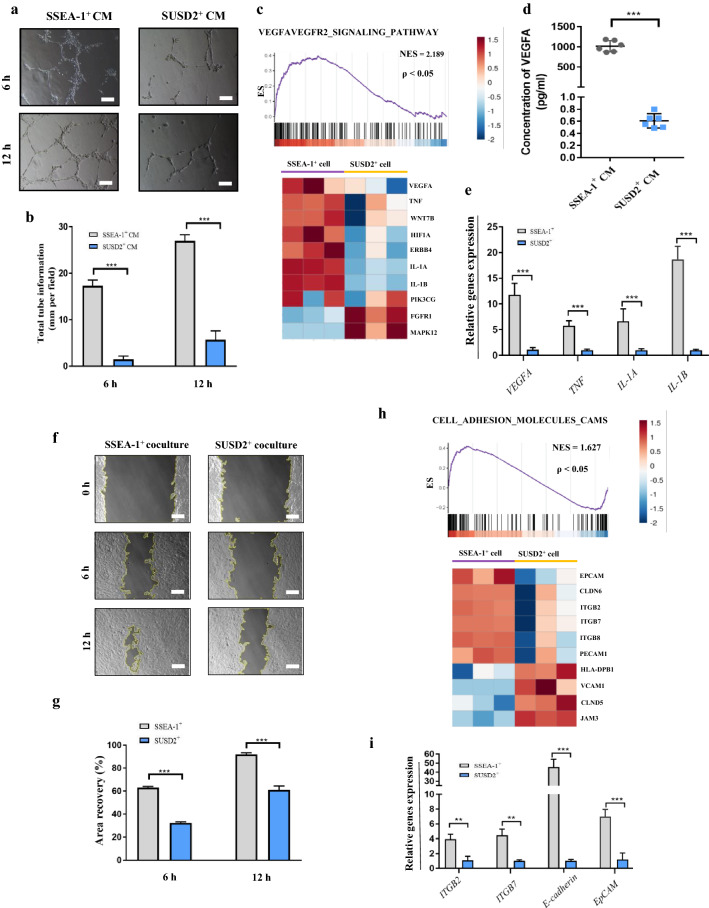
Comparison of functional studies of SSEA-1^+^ and SUSD2^+^ cells. **a** Representative images showing HUVECs tube formation in SSEA-1^+^-CM and SUSD2^+^-CM. CM, conditioned medium. **b** Graph on tube formation for HUVECs incubated with SSEA-1^+^-CM and SUSD2^+^-CM (Error bars represent s.d.; n = 3 technical replicates; two-tailed unpaired t-test, ***P < 0.001). **c** Analysis of gene set enrichment analysis (GSEA) highlighting the upregulation of in SSEA-1^+^ cells (NES = 2.189 of the VEGFA-VEGFR2 signaling pathway). **d** VEGFA in supernatant of SSEA-1^+^ and SUSD2^+^ cell analyzed by enzyme-linked immunosorbent assay (n = 6,*** *P* < 0.001). **e** qPCR analyses for the expression of *VEGFA*, *TNF*, *IL-1A* and *IL-1B* in SUSD2^+^ cells and SUSD2^+^ cells. Expression normalized to β-actin (n = 3 donors, two-tailed unpaired t-test, ***P < 0.001). **f** Representative images of wound healing assay of endometrial stromal cells carried out in SSEA-1^+^ and SUSD2^+^ coculture groups at 0 h, 12 h and 24 h. Scale bars, 100 µm. **g** Bar charts of relative wound healing from the experiments (n = 3 donors, two-tailed unpaired t-test, **P < 0.01, ***P < 0.001). **h** Analysis of GESA revealed the upregulation of cell adhesion in SSEA-1^+^ cells (NES = 1.627 of the cell adhesion molecules cams pathway. The following heatmaps show the subsets of enriched genes involved in their corresponding pathway. (**i**) qPCR analyses for the expression of *ITGB2*, *ITGB7*, *E-cadherin* and *EpCAM* in SSEA-1^+^ cells and SUSD2.^+^ cells, respectively (n = 3 donors, two-tailed unpaired t-test, **P < 0.05, ***P < 0.001)

Angiogenesis plays a critical role in endometrial regeneration. To interrogate the angiogenic capacity of SSEA-1^+^ and SUSD2^+^ cells, the tube-CM (conditioned medium) from both the two cells were collected. The HUVECs exhibited more intensive and extended tubular networks after incubation with SSEA-1^+^-CM (Fig. 5a). The length per tube were significantly higher in SSEA-1^+^-CM treatment than those in SUSD2^+^-CM at both 6 and 12 h (Fig. 5b). In accordance, GSEA demonstrated an enrichment of VEGFA-VEGFR2 signaling pathway-related gene sets in SSEA-1^+^ cells (Fig. 5c). As VEGFA is a vital factor in angiogenesis [41], ELISA was performed to evaluate the VEGFA secretion from SSEA-1^+^ and SUSD2^+^ cells. The results revealed that the concentration of VEGFA were obviously higher in SSEA-1^+^-CM (1016 ± 56.61 pg/ml vs 0.61 ± 0.05 pg/ml, *P* < 0.001, Fig. 5d). Furthermore, the qPCR analysis validated that the SSEA-1^+^ cells displayed higher gene expressions of *VEGFA* and other related transcripts including *TNF*, *IL-1A* and *IL-1B*, than SUSD2^+^ cells (Fig. 5e). These data suggest that SSEA-1^+^ cells had higher angiogenic potential than SUSD2^+^ cells through VEGFA-VEGFR2 signaling pathway.

### Endometrial stromal cells (eSCs) were more migratory when cocultured with SSEA-1^+^ cells

A rapid repair with eSCs around the wound were observed by scanning electron microscopic, which was vital for wound healing of the endometrium [42]. To investigate the impact of SSEA-1^+^ and SUSD2^+^ cells on cell migration, the human eSCs were used for wound healing assay after coculturing with two cells respectively. The eSCs were positive for Vimentin and negative for Cytokeratin by immunocytochemical staining (Additional file 1: Figure S4a). Migration of the eSCs monolayer was monitored over a period of 6 h and 12 h. Higher closure percentages were obtained in eSCs after cocultured with SSEA-1^+^ cells at both 6 h (62.98 ± 0.47% vs 32.38 ± 0.43%, *P* < 0.001) and 12 h (91.85 ± 0.73% vs 60.93 ± 1.71%, *P* < 0.001), than that in SUSD2^+^ cells coculture group (Fig. 5f, g). Meanwhile, GSEA revealed an enrichment of cell adhesion molecules (CAMS)-related gene sets in SSEA-1^+^ cells (Fig. 5h). CAMs enable cells to interact with other cells, influencing a wide variety of fundamental processes like tissue remodeling, repair and regeneration [43]. Next, the qPCR analysis validated that the CAMS related genes (*ITGB2*, *ITGB7*, *E-cadherin* and *EpCAM*) of eSCs were significantly up-regulated in SSEA-1^+^ cells than in SUSD2^+^ cells (Fig. 5i). Taken together, these findings demonstrated that the up-regulated CAMS in SSEA-1^+^ cells might lead to the increased migration of eSCs than SUSD2^+^ cells did in vitro.

### SSEA-1^+^ cells seeded-chitosan implantation boosted endometrial regeneration of IUA rats

We established the IUA rat models via a scraping treatment to mimic the histopathological changes in IUA patients [44]. The uteri were collected 14 days after endometrial damage for gross, histological, and fibrosis evaluation. The IUA rat model was confirmed to have thinner endometrium, fewer endometrial glands and more fibrosis area than the normal endometrial cavity (Additional file 1: Figure S5a to e).

Chitosan is synthetic high molecular polysaccharide substance that poses great biocompatibility, degradability and biological activity, which can conduct as a physical barrier and inhibit the formation of accumulated scar tissue. All these properties make chitosan increasingly popular for many biomedical applications such as tissue engineering and regenerative therapies [45]. Thus, we further investigated the in vivo therapeutic effects of SSEA-1^+^ and SUSD2^+^ cells/chitosan on IUA rats by dividing the 20 rats into four groups: chitosan-saline solution group (chitosan group), SUSD2^+^ cells-laden chitosan group (SUSD2^+^ group), SSEA-1^+^ cells-laden chitosan group (SSEA-1^+^ group) and combination of SUSD2^+^ and SSEA-1^+^ cells-laden chitosan group (SSEA-1^+^  + SUSD2^+^ group). The left uteri of each rat received endometrial scraping while the contralateral right uteri were kept as control. The control group received no intervention. Schematic illustration of the experimental procedures was shown in Additional file 1: Figure S5f. As shown in Additional file 1: Figure S6, at day 14 post-surgery, severe hydrometra and structural deformation of the wound uteri were observed in all rats with only chitosan-saline solution treatment. In the SUSD2^+^ group, although one rat was found with a normal-seeming uterus, the others were still found to be abnormal with a mild to moderate hydrometra. By contrast, normal-seeming uteri were harvested in the SSEA-1^+^ group and the combination groups.

**Fig. 6:**
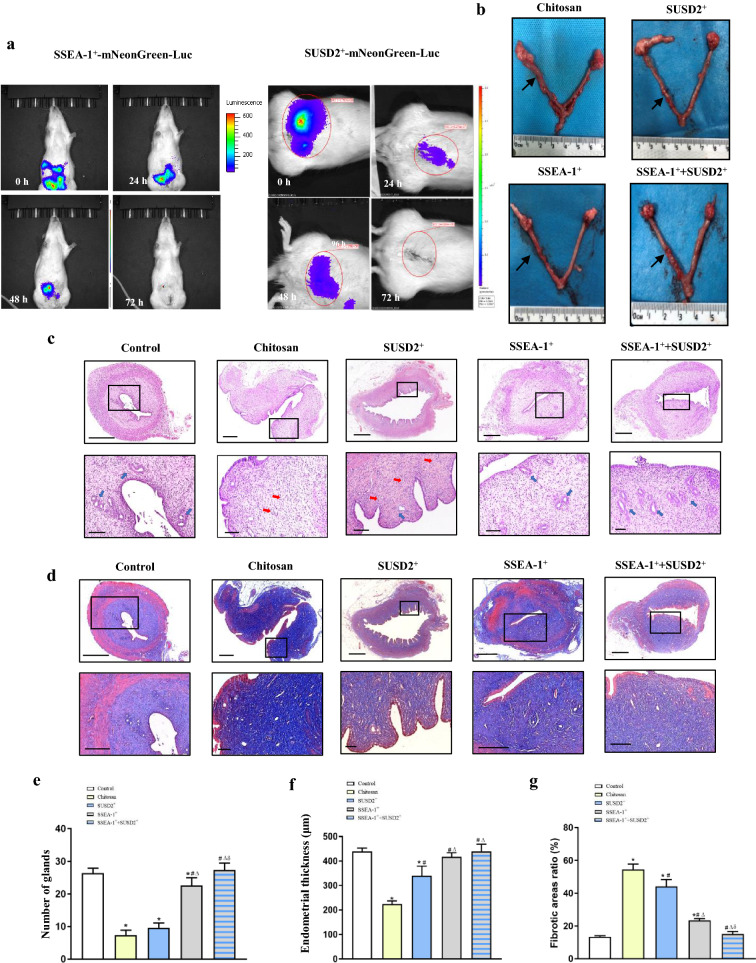
The regenerative uterine horns after chitosan and cell therapy. **a** Fluorescent images of rats after transplantation with SSEA-1^+^ or SUSD2^+^ cells-laden chitosan. **b** Representative morphology of uteri following different treatments for 14 days. **c** HE staining of uteri after different treatments for 14 days. Blue arrow, intact endometrial glands; red arrow, incomplete endometrial gland. Inserted overview pictures are of lower magnification (scale bars, 500 µm); black squares are highly magnified regions (scale bars, 200 µm). **d** Collagen staining of uteri using Masson trichrome after different treatments for 14 days. Inserted overview pictures are of lower magnification (scale bars, 500 µm); black squares are highly magnified regions (scale bars, 200 µm). **e–g** Statistical analysis of the number of glands **e**, the endometrial thickness **f** and the percentages of collagen positive staining **g** after different treatments. LSD method was used for pairwise comparison between groups, n = 5, *P < 0.05, compared to the Control group; #*P* < 0.05, compared to the Chitosan group; Δ*P* < 0.05, compared to the SUSD2^+^ group; &*P* < 0.05, compared to the SSEA1^+^ group

Restoration of the endometrium following different interventions was assessed by HE staining. The chitosan group and SUSD2^+^ group exhibited endometrial regeneration with few or no intact luminal structures (Fig. 6c). Endometrial gland numbers and endometrial thickness decreased markedly in the chitosan group (7.4 ± 2.1; 225 ± 13 μm) and SUSD2^+^ group (9.6 ± 1.5; 341 ± 39 μm), as compared with those in the control group (26.4 ± 2.7, *P* < 0.05; 441 ± 13 μm, *P* < 0.05, Fig. 6e,f). By contrast, the structure of the endometrium appeared well organized with more luminal intact glands in the stromal layer and the thickness of endometrium increased significantly in the SSEA-1^+^ group when compared with the normal uteri (22.6 ± 2.4,* P* < 0.05; 417 ± 17 μm, *P* > 0.05). Furthermore, these results were more pronounced in the combination group, as compared to the control group (27.4 ± 2.1,* P* > 0.05; 440 ± 29 μm, *P* > 0.05, Fig. 6c, e, f).

To evaluate collagen remodeling in the reconstructed endometrium after transplanting SSEA-1^+^ and SUSD2^+^ cells/chitosan, Masson’s trichrome staining was performed (Fig. 6d) and fibrotic areas were analyzed quantitatively (Fig. 6g). At day 14 post-transplantation, more collagen deposition was observed in the chitosan group than that in the control group (13.4 ± 0.7% vs. 54.6 ± 3.3%,* P* < 0.05). A significantly decreased degree of fibrotic area was found in the SUSD2^+^ group when compared with the normal uteri (44.2 ± 4.2%,* P* < 0.05). Furthermore, a much greater reduction in fibrotic area was found in the SSEA-1^+^ group, as compared to the control group (23.4 ± 1.2%,* P* > 0.05). Remarkably, the collagen deposition of the combination group was similar to that of the control group, suggesting that the SSEA-1^+^ cells-laden chitosan injection significantly reduced fibrosis after the damage to the endometrium.

The survival of the transplanted cells in vivo is critical to improve the endometrial regeneration. Therefore, we next evaluated the effect of chitosan on the survival of the transplanted cells in vivo. We infected SSEA-1^+^ and SUSD2^+^ cells with luciferase by lentivirus for in vivo fluorescent imaging of transplanted cells. The representative morphology of SSEA-1^+^- and SUSD2^+^-mNeonGreen-Luc cells was shown in Additional file 1: Figure S4c,e. The fluorescent image of SSEA-1^+^ cells was presented in Additional file 1: Figure S4d. The activity of luciferase of SUSD2^+^ cells and SUSD ^+^ cells transduced with lentivirus in vitro was also detected by D-Luciferin sodium in vitro (Additional file 1: Figure S4f). The activity of luciferase in vivo was detected after operation and the results were presented in Fig. 6a. The results revealed that the survival time of SSEA-1^+^ and SUSD2^+^-mNeonGreen-Luc was approximately 48 h.

## Discussion

Two important outcomes of our study are the establishment of a novel approach for isolation and expansion of human endometrial epithelial progenitor-like cells, or SSEA-1^+^ cells in vitro and a regenerative medicine-based treatment to IUA in a rat model. So far as we know, this is the first report to show that human endometrial SSEA-1^+^ cells can be efficiently isolated and expanded in vitro. These cells exhibited strong adult stem cell properties and high proliferative potential with human SUSD2^+^ eMSCs as a control. The SSEA-1^+^ cells satisfied most of the phenotypic characteristics of eESCs: they were expanded from endometrial EpCAM^+^ cells; they showed great clonal capacity in vitro; they could generate organoids and could differentiate to EEC-like cells with a high efficiency; and the transcriptome sequencing results indicated that they expressed lineage-related genes in the processes of epidermis development and keratinocyte differentiation. Thus, our data provide strong in vitro evidence which suggests that the cultured SSEA-1^+^ cells are the endometrial epithelial progenitor-like cells.

The early studies on isolation of human eESCs were based on their clone forming ability and did not allow us to prospectively isolate them from endometrium [2, 5]. After a long period of exploration of identifying the marker of human eESCs, the SSEA-1^+^ cells were most likely to be the putative eESCs [10, 46, 47]. In the present study, we used SSEA-1 as the marker to purify postulated eESCs. With a cocktail of small molecules, the purified SSEA-1^+^ cells in long-term culture exhibited great clonal capacity and could maintain their polarity as monolayers in vitro. In addition, SSEA-1^+^ cells could differentiate to EEC-like cells which expressed classic epithelial markers (EpCAM and CD9). The reduced expression of pluripotent genes *OCT-4*, *SOX-2* and *Nanog* and the increased expression of somatic genes *EpCAM* and *CD9* were observed. It suggested that the SSEA-1^+^ cells had switched from a progenitor stage to a mature stage during differentiation. Moreover, the SSEA-1^+^ organoids expressed classic epithelial markers including Cytokeratin and ER, which reflect the epithelial compartment of endometrial tissue. Indeed, although the human organoids were believed to be initiated from the stem cells of tissues, the human endometrial organoids were mostly developed from endometrial tissues. The recalcitrant to robust expansion of endometrial epithelial cells becomes an obstacle. Thanks to the long-term maintenance of SSEA-1^+^ cells, we achieved the development of long-term expandable organoids from pure human eESCs, instead of fragments or co-culturing systems. Consistent with the previous studies, the SSEA-1^+^ cells not only had strong clonal capacity and great efficiency of differentiation to EEC-like cells, but also had organoids-forming ability, which indicates that SSEA-1 is an appropriate single marker for identifying eESCs.

To further understand the function of SSEA-1^+^ cells, the differences in transcriptome‐wide gene expression between SSEA-1^+^ and SUSD2^+^ cells were analyzed. The differentially expressed genes of SSEA-1^+^ cells were mostly enriched in cytokine activity, receptor activity, cell-cell junction, epidermis development and keratinocyte differentiation. These pathways play important parts in modulating the regeneration of endometrial epithelium by SSEA-1^+^ cells as proposed by Valentijn et al. [10]. On the other hand, the characterization of the SUSD2^+^ cells we achieved was consistent with human eMSCs isolated from menstrual blood or endometrial tissue [7, 23, 48]. Furthermore, the GO enrichment analysis revealed that SUSD2^+^ cells expressed more genes in regulating mesenchyme development and extracellular matrix component. That added another evidence that eMSCs regulates human endometrial stromal regeneration.

According to the mainstream hypothesis, the human endometrial somatic stem/progenitor cells are responsible for maintaining the endometrium homeostasis [49–52]. Cervelló et al. had generated endometrial-like tissue when both epithelial and stromal side population cells were injected together, and only epithelium was formed when the epithelial side population cells were transplanted [53]. Another in vitro study had formed a gland-like structures with SSEA-1^+^ cells in 3D culture, and its unipotency was further improved by forming endometrial gland-like structures in an animal model [10, 54]. We also generated glandular organoids with long-term cultured SSEA-1^+^ cells that resembled other human organoids derived from different adult epithelial stem cells [34, 55, 56]. With the findings of this study, we believe that the re-epithelialization of endometrium was efficiently imitated by SSEA-1^+^ cells. However, how SSEA-1^+^ cells were involved in endometrial regeneration is an intriguing question and needs to be further investigated. Different SSEA-1^+^ cells subsets, such as SSEA-1^+^/N-cadherin^+^ and SSEA-1^+^/N-cadherin^−^ cells, were found in endometrial tissues [9, 57]. The TEM system we used can culture progenitor cells and may even be able to reprogram terminally somatic cells to a proliferative progenitor like state [15, 17]. That indicated the SSEA-1^+^ cells we purified might be expanded from the original SSEA-1^+^ cells or be reprogrammed from the epithelial cells. A recent study demonstrated that the self-renewal activity of eMSCs is dynamically changing during the menstrual cycles [58]. Thus, an alternative or additive mechanism of regeneration theory is that the epithelial and stromal cells could dedifferentiate into a proliferative progenitor like state with a capacity for proliferation and differentiation.

The repair of endometrium after trauma or injury, unlike the physiological repair in menstrual cycle, may end up with endometrial fibrosis or adhesion due to the imbalance of stromal-epithelial crosstalk [59]. In recent years, stem cell therapy has been recognized as a potential treatment strategy for IUA. Therefore, we evaluated the therapeutic effect of the endometrial stem cells in a rat model of IUA. Although two rats with SUSD2^+^ cells-laden chitosan treatment showed a slight improvement, the others ended up as severe as control. However, with the treatment of SSEA-1^+^ cells, the injured endometrial tissue presented very minimal fibrosis with a normal uterus and significant glandular proliferation. VEGFA is a key mediator of angiogenesis by which re-epithelialization of the endometrium is improved [60–62]. Our data showed that, in comparison with SUSD2^+^ cells, SSEA-1^+^ cells promoted tube formation with higher secretion of VEGFA, and this was accompanied by increased expression of genes related to VEGFA-VEGFR2 signaling pathway. It indicates that the angiogenic effect of SSEA-1^+^ cells might facilitate the reconstruction of microvessels and endometrial tissue. Furthermore, according to Garry et al., the healing endometrial surface is firstly covered with a fibrinous matrix and then the new surface epithelium develops [42]. In support of this notion, the wound healing assay revealed that coculture with SSEA-1^+^ cells led to increased eSCs migration in vitro. Consistent with this observation, the SSEA-1^+^ cells expressed more genes related to CAMS pathway. In addition, the chitosan we used is a synthetic high molecular polysaccharide substance that poses natural origin and several biological properties: biocompatibility, non-toxicity, non-allergenicity, and biodegradability [63]. At present, chitosan has been frequently used to prevent re-adhesion after abdominal surgery [64]. Fluorescent imaging of the rats suggested that the present stem cells-laden chitosan can be evenly dispersed by the contraction of the uterine cavity and work as a physical barrier to block damaged wounds. Furthermore, the stem cells embedded in chitosan could be retained in the uterine cavity for approximately 48 h, which may promote the regeneration of the uterus in early repair stages. Together, these data collectively demonstrated that SSEA-1^+^ cells might urge endometrial stromal cells to cover the wound endometrium surface and facilitate angiogenesis by a paracrine mechanism, accompanying with the separation effect of chitosan, which could be the primary cause for improved endometrium restoration in this IUA model.

Last but not the least, we found that after MACS- and FACS-based sorting, the purified SSEA-1^+^ cells exhibited remarkable phenotypic homogeneity. The similar phenotypic homogeneity was also found on the SUSD2^+^ cells. Furthermore, the cell purity was not affected by the sample variation from different individuals. It indicates that we could readily generate patient-specific eESCs and eMSCs from minimally invasive hysterectomy or endometrial biopsy samples. Our findings provide a potential platform for producing a feasible cell source to explore the mechanism of endometrial regeneration and a promising pathway towards endometrial stem cell therapy.

In summary, this is to our knowledge the first report demonstrating a method for long-term in vitro culturing and expansion of human endometrial SSEA-1^+^ cells that manifest phenotypic properties of endometrial epithelial progenitor-like cells. Notably, our findings indicate a role of SSEA-1^+^ cells in regulating angiogenesis and eSCs migration during endometrial regeneration. Also, the in situ endometrial stem cells-laden chitosan transplantation provides a novel strategy for endometrial regeneration in IUA rats. Further insights into the cellular interactions between SSEA-1^+^ and SUSD2^+^ cells will be invaluable for understanding pathophysiology of endometrial restoration, ultimately leading to more rational clinic practice.

## Materials and methods

### Human primary endometrial EpCAM^+^ and EpCAM^−^ cells isolation and purification

Human endometrium samples were obtained from the Shanghai Ji Ai Genetics & IVF Institute. Patients who underwent hysterectomy or endometrial biopsy for nonendometrial benign pathologies were qualifying candidates. None of the donors had taken exogenous hormones for at least 3 months prior to surgery (See Supplementary information, Additional file 1: Table S3 for detailed information). This study was approved by the Ethics Committee of the Shanghai Ji Ai Genetics & IVF Institute, and informed written consent was obtained from each patient. Fresh endometrium samples were collected on the 11th or the 12th day of the menses, and the samples were mixed with triple volume of DMEM F/12 containing 5% FBS (Gibco, USA) and 3% antibiotic–antimycotic mixture. The endometrial tissue was dissociated into single-cell suspensions using 1% collagenase type I (diluted in 0.25% trypsin/1 mM EDTA; BioLegend, USA) at 37 °C for 60 min. The 100 µm cell strainer was used to remove the remain tissue pieces and the collected cells were treated with red blood cell lysis buffer. Purified EpCAM^+^ and EpCAM^−^ cell suspensions were then obtained by magnetic bead sorting using EasySep Human EpCAM Positive Selection Kit (BioLegend, USA). The positive and negative selections were incubated with PerCP-conjugated anti-human EpCAM antibody (BioLegend, USA) and analyzed by flow cytometry (Additional file 1: Figure S1a).

### Cell expansion

Purified human endometrial EpCAM^+^ and EpCAM^−^ cells were seeded onto 12-well plates and cultured in TEM. This was based on DMEM/F12 (Invitrogen) supplemented with ITS or N2 (both from Invitrogen), and the following factors: 20 ng/ml EGF (Peprotech), 10 μM Y27632, 3 μM CHIR99021, 1 μM A8301 (all from TargetMol), 1 μM S1P and 5 μM LPA (both from Santa Cruz). TEM was changed every 2 days. When the cells reaching 80–90% confluence, clonal cells were passaged at a ratio of 1:3 after dissociation with 0.25% trypsin/1 mM EDTA. To assess population doubling time, 10,000 cells were seeded on 24-well plates and counted on designated days, following dissociation with 0.25% trypsin/1 mM EDTA to obtain single cells. Population doubling time was calculated as following: log2/ (logNt – logN0), t = time period (h), Nt = number of cells at time t, N0 = initial cell number.

### Karyotyping analysis

The SSEA-1^+^ and SUSD2^+^ cells at the 10^th^ passage in exponential growing phase were incubated with 100 ng/mL colcemid for 40 min at 37 °C. Cultures were then washed and dissociated into single cells using 0.25% trypsin/1 mM EDTA. Karyotyping was performed at the karyotype analysis department of the Shanghai Ji Ai Genetics & IVF Institute. Chromosomes from at least 40 metaphase-arrested cells were counted.

### Fluorescence-activated cell sorting (FACS) of SSEA-1^+^ cells

At the 3rd passages, FACS were performed using MoFlo XDP (Beckman, USA). Briefly, the 3rd -passage-EpCAM^+^ cells were digested with 0.25% trypsin/1 mM EDTA and washed with Stain Buffer (BD Biosciences, USA). The detached cells were incubated with the APC-conjugated anti-human CD15 antibody (BioLegend, USA) in Stain Buffer containing 3% antibiotic–antimycotic mixture at a 1:20 dilution. The sorted SSEA-1^+^ cells were collected in TEM containing 3% antibiotic–antimycotic mixture.

### Flow cytometry

Selected cell surface markers were characterized by flow cytometry (BD LSRFortessa). Data were analyzed by FlowJo software (Ashland, OR, USA). Cells were detached with 0.25% trypsin/1 mM EDTA, and then washed with Stain Buffer. The detached cells were incubated with the labeled primary antibodies (listed in supplementary information, Additional file 1: Table S2) at 4 °C for 30 min. Then, the cells were washed with Stain Buffer and were ready for analysis. The FACS gates were established by staining isotype antibodies or blank controls.

### Cell counting kit-8 (CCK-8) assay

The SSEA-1^+^ and SUSD2^+^ cells were suspended and seeded onto 96-well plates at 4 × 10^3^ cells per well. CCK-8 reagent (10 μl; Beyotime Biotechnology, China) was added into each group after being cultured for 24, 48, 72, 96, 120 and 144 h. After incubation for 2 h at 37 °C, the proliferation was determined by absorbance measurement at 450 nm using ELX800 Universal Microplate Reader (BioTek, USA).

### Colony formation assay

The SSEA-1^+^ and SUSD2^+^ cells at the logarithmic growth phase were seeded onto 6-well plates (1 × 10^3^ cells/well), followed by 14 days culture with TEM until cell colonies were visible. Cells were fix with 4% paraformaldehyde for 20 min and stained with crystal violet staining solution for 20 min. The number of clusters with more than 50 cells were counted.

### Detection of the apoptotic rate

P1 and P10 SSEA-1^+^ cells at a density of 1 × 10^5^ cells/ml were inoculated into 6‑well cell culture plates and incubated at 37˚C for 4 days, until the bottom of the plates were covered. Cell apoptosis was detected according to the instructions of an Annexin V‑FITC apoptosis detection kit (BD Pharmingen, USA).

### Immunofluorescence staining

The SSEA-1^+^ and SUSD2^+^ cells at the logarithmic growth phase were seeded onto u-Slide 8-well plate (IBIDI, Germany) at 2 × 10^3^ cells per well, cultured in TEM. When the cells reaching 80% confluence, both SSEA-1^+^ and SUSD2^+^ cells were fixed with 4% paraformaldehyde for 20 min. For SSEA-1^+^ cells, permeabilization was performed using 0.05% Triton-X 100 for 10 min. The cells were blocked with 30% donkey serum for 30 min at room temperature and then incubated with the primary antibodies (anti-SSEA-1 and anti SUSD2, Abcam, USA, respectively) over night at 4 °C overnight. Next, the cells were incubated with the secondary donkey anti-rabbit or anti-mouse antibody for 1 h at room temperature. Then, cells were counterstained with Hoechst 33,342 (Sigma, USA) to identify all nuclei. Images were captured with an A1 + inverted confocal microscope (Nikon, Japan).

### Establishment of endometrial organoids

1–2 × 10^5^ P3 SSEA-1^+^ or SUSD2^+^ cells were resuspended in 100 μl thawed Matrigel membrane (BD Biosciences, USA) and placed in drops on the bottom of 48-well plates. Then, the plates were inverted and placed in the 37 °C incubator for 20 min to allow gelation. After solidification of cell-Matrigel mixture, the plates were added with 1 ml/well TEM to cover the Matrigel and the TEM was changed every 3–4 days. To obtain intact spheres for passing or immunofluorescence staining, 1 ml/well precooled Cultrex Organoid Harvesting Solution (R&D Systems, USA) was added and incubated on ice for 40 min. Spheres were centrifuged at 300 g for 5 min and washed wish PBS to remove the depolymerized Matrigel. For suspension culture, 1–2 × 10^6^ P3 SSEA-1^+^ or SUSD2^+^ cells were seeded in the low-attachment 6-well plate. The SUSD2^+^ cells began aggregation within 6 h and formed a solid and non-opaque spheres after 24 h in TEM. Hollow and translucent organoids were observed in SSEA-1^+^ cells after 24 h in TEM.

### Immunofluorescence staining of spheroids

The spheroids were fixed with 4% paraformaldehyde for 20 min and blocked using 5% BSA for 60 min. Following blocking, the spheroids were incubated at 4 °C overnight with primary antibodies diluted in 1% BSA. The spheroids were incubated with the respective secondary antibodies for 1 h at room temperature. Then, spheroids were counterstained with Hoechst 33,342. Images were captured with an A1 + inverted confocal microscope.

### Differentiation of SSEA-1^+^ and SUSD2^+^ cells into endometrial epithelial cell-like (EEC-like) cells

P3 SSEA-1^+^ and SUSD2^+^ cells were seeded onto six-well plate at a density of 6 × 10^5^ cells/well in TEM. When reached 90% confluence, cells were cultured in differentiation medium consisting of DMEM/F12 with 5% FBS, 1 × 10^–6^ mol/L Estradiol (E2) (Absin, China), 10 ng/ml recombinant human epidermal growth factor (EGF) (R&D Systems, USA), and 10 ng/ml recombinant human platelet-derived growth factor-BB (R&D Systems, USA). The differentiation medium was changed every 2 days for a 20-day differentiation (Fig. 3b). Cytokeratin and Vimentin were tested through immunofluorescence staining. Anti-CD9 and anti-EpCAM were used to detect the results of the EEC-like cells differentiation by flow cytometry. The expressions of pluripotency and epithelium related genes of SSEA-1^+^ and SUSD2^+^ cells before and after differentiation were measured by qPCR analysis.

### Preparation of conditioned medium (CM)

The SSEA-1^+^ and SUSD2^+^ cells were seeded onto 6-well plates at a density of 6 × 10^5^ cells/well. After 72 h, when cells reached approximately 80% confluence, the TEM was changed every 24 h. Then, the supernatants were harvested as CM for use in next experiments. For tube formation assay, when cells reached approximately 80% confluence, the TEM was replaced with Endothelial Cell Medium (ScienCell, USA). The medium was changed every 24 h and supernatants were harvested as tube-CM for tube formation assay. The supernatants were collected, pooled, centrifuged at 1000 g, and stored at −80 °C.

### Tube formation assay

Human umbilical vein endothelial cells (HUVECs) were obtained from ScienCell (USA) and cultured in Endothelial Cell Medium. When reached 80% confluence, the HUVECs were seeded onto 96-well plates coated with Matrigel membrane in the presence of CM from SSEA-1^+^ and SUSD2^+^ cells. Tube formation was visualized under light microscope (Olympus) at 6 and 12 h after incubation. Number of branches was counted for analysis.

### Enzyme-linked immunosorbent assay (ELISA)

ELISA was performed to compare the secretion of VEGFA from SSEA-1^+^ and SUSD2^+^ cells. The VEGFA concentrations were determined using a Human VEGFA ELISA kit (R&D Systems, USA) according to the manufacturer’s instructions. The absorbance of the samples at 450 nm using ELX800 Universal Microplate Reader.

### Wound healing assay

Primary human endometrial stromal cells (eSCs) were isolated basing on a modification of the method of Satyaswaroop and colleagues [65]. In brief, the endometrial tissue was dissociated into single-cell suspensions using collagenase type I (diluted in 0.25% trypsin/1 mM EDTA). Then, the cell suspensions were filtered using a 100 µm sieve to separate single cells from undigested tissue fragments, and a 40 µm sieve to separate epithelial glands. The eSCs passed through to the below and were cultured in medium consisting of DMEM/F12 with 10% FBS. The eSCs were then stained with anti-Cytokeratin and anti-Vimentin antibodies and identified by immunohistochemistry. The P3 eSCs were cultured in a transwell system (hanging cell culture insert with 1 µm pore polycarbonate membrane in 6-well plate; Millipore sigma, German). In brief, the eSCs were cultured in the bottom of a 6-well plate in DMEM/F12 with 10% FBS and the SSEA-1^+^ and SUSD2^+^ cells were seeded on the transwell membrane at a density of 6 × 10^5^ cells/well in TEM. When the cells grew to 90% confluence, eSCs were cocultured with SSEA-1^+^ and SUSD2^+^ cells in TEM for 24 h respectively. Then, an injury line was made using a 10-µl plastic pipette tip and the cells were kept being cocultured with SSEA-1^+^ and SUSD2^+^ cells for another 24 h. The migration distance was measured at 0 h, 12 h and 24 h after scratching.

### Quantitative real-time polymerase chain reaction (qPCR)

Total RNA of cells and rat tissues were extracted using TRIzol reagent according to the manufacturer’s protocols. The quality and quantity of the total RNA were checked by spectrophotometry. The RNA was reverse transcribed by the high-capacity complementary DNA reverse transcription kit (Roche). qPCR analyses were performed using a LightCycler^®^ 96 Real-Time PCR System (Roche) and SYBR Green PCR kit (Roche). Gene transcription was evaluated using the ΔΔCt method normalized to *β-actin*. Primer sequences are respectively listed in supplementary information, Additional file 1: Table S1.

Detailed protocols for RNA-sequencing analysis, the procedures and evaluation of animal experiments, the study of eSCs and SUSD2^+^ cells cocultured with SSEA-1^+^ cells in vitro can be found in Supplementary Methods.

### Statistical analysis

Results were expressed as the means ± SD and the analyses were performed using SPSS software version 22.0. Two samples were compared using independent sample *t* test. One-way analysis of variance (ANOVA) was utilized to determine significant differences between multiple groups and the LSD method was used for pairwise comparison between groups. *P* < 0.05 was considered as statistically significant.

## Supplementary Information

**Additional file1: Figure S1.** Phenotypic characterization of human SSEA-1^+^ cells. **Figure S2.** Isolation and culture human SSEA-1^+^ cells, related to Figure 1. **Figure S3.** Individual variations in proliferative potential of human SSEA-1^+^ and SUSD2^+^ cells from different donors. **Figure S4.** The identification of eSCs, P0 endometrial cells, SSEA-1^+^ and SUSD2^+^ cells transduced with lentiviral vector expressing mNeonGreen and luciferase. **Figure S5.** The uterine horns at day 14 after injury. **Figure S6.** Morphology of uteri in four groups following different treatments for 14 days. **Table S1.** Primers used for q-PCR. **Table S2.** Antibody list. **Table S3.** Donor information.

**Additional file 2. **3D confocal scanning images of the SSEA-1^+^ cells.

**Additional file 3. **3D confocal scanning images of the SUSD2^+^ cells.
