# Supplementary material for: Long-term maintenance of human endometrial epithelial stem cells and their therapeutic effects on intrauterine adhesion
Source: Cell Biosci. 2022 Oct 18;12:175. doi: 10.1186/s13578-022-00905-4 (PMC9580151; doi:10.1186/s13578-022-00905-4)
Supplement: Supplementary file 1 — Additional file1: Figure S1. Phenotypic characterization of human SSEA-1+ cells. Figure S2. Isolation and culture human SSEA-1+ cells, related to Figure 1. Figure S3. Individual variations in proliferative potential of human SSEA-1+ and SUSD2+ cells from different donors. Figure S4. The identification of eSCs, P0 endometrial cells, SSEA-1+ and SUSD2+ cells transduced with lentiviral vector expressing mNeonGreen and luciferase. Figure S5. The uterine horns at day 14 after injury. Figure S6. Morphology of uteri in four groups following different treatments for 14 days. Table S1. Primers used for q-PCR. Table S2. Antibody list. Table S3. Donor information. [file 13578_2022_905_MOESM1_ESM.docx]

**Long-term maintenance of human endometrial epithelial stem cells and their therapeutic effects on intrauterine adhesion**

Wen He^1,†^, Xuejing Zhu^2,†^, Aijie Xin^3,†^, Hongdan Zhang^2^, Yiming Sun^1^, Hua Xu^4^, He Li^4^, Tianying Yang^1^, Dan Zhou^1^, Hexin Yan^2,*^, Xiaoxi Sun^1,4,5,*^

*^1^Obstetrics and Gynecology Hospital, Fudan University, Shanghai, China.*

*^2^Shanghai Celliver Biotechnology Co. Ltd., Shanghai, China.*

*^3^NHC Key Lab of Reproduction Regulation (**Shanghai Institute for Biomedical and Pharmaceutical Technologies), Fudan University, Shanghai, China.*

*^4^Shanghai Ji Ai Genetics and IVF Institute, Obstetrics and Gynecology Hospital of Fudan University , Shanghai, China.*

*^5^Shanghai Key Laboratory of Female Reproductive Endocrine Related Diseases, Shanghai, China.*

^†^These authors contributed equally to this work.

*Correspondence: [xiaoxi_sun@aliyun.com](mailto:xiaoxi_sun@aliyun.com) (X.-X.S.); [hexin.yan@celliver.com](mailto:hexin.yan@celliver.com) (H.-X.Y.)

**Methods**

## RNA-seq and bioinformatics analysis

For RNA-seq, the total RNA was isolated from the SSEA-1^+^ and SUSD2^+^ cells from three donors using TRIzol reagent (Invitrogen) according to the reagent protocols. The quality and amount of RNA were determined using the NanoPhotometer® spectrophotometer (IMPLEN, CA, USA) and Agilent 2100 Bioanalyzer (Agilent, CA, USA). NEBNext® UltraTM RNA Library Prep Kit was used for library preparation. After libraries were prepared, the samples were sequenced on an Illumina Novaseq platform. Raw data were cleaned by removing reads containing adapter, reads containing ploy-N and low-quality reads. All the downstream analyses were based on the clean data with high quality. FeatureCounts v1.5.0-p3 was used to count the reads numbers mapped to each gene. And then FPKM (fragments per kilobase of exon per million mapped reads) of each gene was calculated based on the length of the gene and reads count mapped to this gene. Differential expression analysis of two conditions/groups (two biological replicates per condition) was performed using the DESeq2 R package (1.16.1). Corrected *P*-value of 0.05 and absolute foldchange of 2 were set as the threshold for significantly differential expression. Gene Ontology (GO) enrichment analysis of differentially expressed genes was implemented by the cluster Profiler R package, in which gene length bias was corrected. GO terms with corrected *P* value less than 0.05 were considered significantly enriched by differential expressed genes. Gene set enrichment analyses (GSEA) were performed using the GSEA software.

## Experimental animals

All experiments were performed with female Sprague-Dawley rats of 8 weeks (Shanghai JieSiJie Laboratory Animals Co,.LTD, China). Rats were housed in an air-conditioned animal house with free access to food and water. All experimental procedures with animals were performed according to the Institutional Guidelines for the Care and Use of Laboratory Animals. All protocols were approved by the institutional review board for animal experiments of the Shanghai Institute for Biomedical and Pharmaceutical Technologies. All researchers involved in animal experiments possessed animal experimentation licenses issued by the Shanghai Lab.Animal Research Center.

## Establishment of IUA rat models and the transplantation procedures

Rats weighted 200-250 g were used to construct the experimental model. After the rats were anesthetized via isoflurane inhalation, the uterus horns were exposed through a low abdominal midline incision. Next, a 1.5 cm longitudinal incision was made on the right side of the uterus to expose the inner endometrium. The endometrium was scraped using a T10 scalpel blade until surface of the uterus was rough and bleeding. The uterus was stitched using 6-0 sutures. After injury, each group received a different treatment with syringe directly injecting into the uterus cavity through the previous incision: 1) Chitosan group: the right side of uteri received only 1 ml 3% Chitosan -saline solution. 2,3) SSEA-1^+^ group and SUSD2^+^ group: the right side of uteri received 1 ml 3% Chitosan (Sigma-Aldrich, USA) -saline ladened with 10^6^ of each cell respectly. 4) SSEA-1^+^ + SUSD2^+^ group: the right side of uteri received 1 ml 3% Chitosan-saline ladened with 10^6^ of both SSEA-1^+^ and SUSD2^+^ cells (schematic illustration was shown in Figure S5F). All rats (n = 20) were randomized into the 4 groups. 4-0 sutures were used to close the incisions on the abdomen. Schematic illustration of experimental procedures was shown in Figure S5F.

## Cell infection

Lentivirus carrying pLenti-CBh-3xFLAG-Luc2-tCMV-mNeonGreen-F2A-Puro-WPRE was purchased from OBio Technology Company (China) to construct SSEA-1^+^ and SUSD2^+^ cells stably expressing luciferase. Cells were seeded in 6-well plates at a density of 6 × 10^5^ cells/well for 24 h, then 1 ml/well prewarmed TEM with Lentivirus in different multiplicity of infection (MOI) was added. Medium was changed with 2 ml/well TEM 24 h later. The expression of mNeonGreen was observed through fluorescence microscope. The SUSD2^+^-Luc cells were selected by puromycin for 3 to 5 days. The expression of luciferase was determined by multifunction microplate reader after D-Luciferin sodium salt was added.

To verify the survival time of SSEA-1^+^ cells, we performed lentiviral transfection when the cells were 70%-80% confluent. The SSEA-1^+^-Luc cells were selected by puromycin (0.5 μg/ml) for 3 to 5 days. The SSEA-1^+^-mNeonGreen-Luc cells and SSEA-1^+^-mNeonGreen-Luc organoids were confirmed by fluorescence microscope and luminescence analyses.

## In vivo fluorescence imaging

The SD rats weighted 200-250 g were used to perform in vivo fluorescence imaging experiment. The methods were as same as the procedures mentioned before except using the SUSD2^+^-Luc cells. The rats were anesthetized with anesthetic and injected intraperitoneally with 5mg D-Luciferin sodium salt (diluted in 200 μl PBS). Fluorescence images were taken by an IVIS Lumina XRMS Series III o imaging system (Caliper LifeSicence, USA) within 10 min.

## Histological analysis

At day 14 post surgery, rats were sacrificed, and their uteri were embedded in paraffin after fixing with 4% paraformaldehyde overnight. Sections were cut into 5 μm and stained with Hematoxylin-eosin Staining to observe the tissue structure and the number of glands. And slides were stained with Masson’s Trichrome to determine the relative content of collagen/connective tissue and smooth muscle in the uterine tissue. Nikon Eclipse 50i (Nikon, Japan) was used to image the sections.

## Histological analysis

The expression of SSEA-1, Cytokeratin Vimentin, ER, and N-cadherin proteins were detected by immunohistochemistry. Slides of human endometrium and SSEA-1^+^ cells forming organoids were incubated with primary antibodies (Table S2) overnight at 4°C, followed by further incubation with horseradish peroxidase (HRP)-conjugated goat anti-mouse IgG or goat anti-rabbit IgG (Abcam, USA). Immunoreactivity was detected using diaminobenzidine (DAB). Pictures were taken by Nikon Eclipse 50i.

**SUPPLEMENTAL FIGURES**


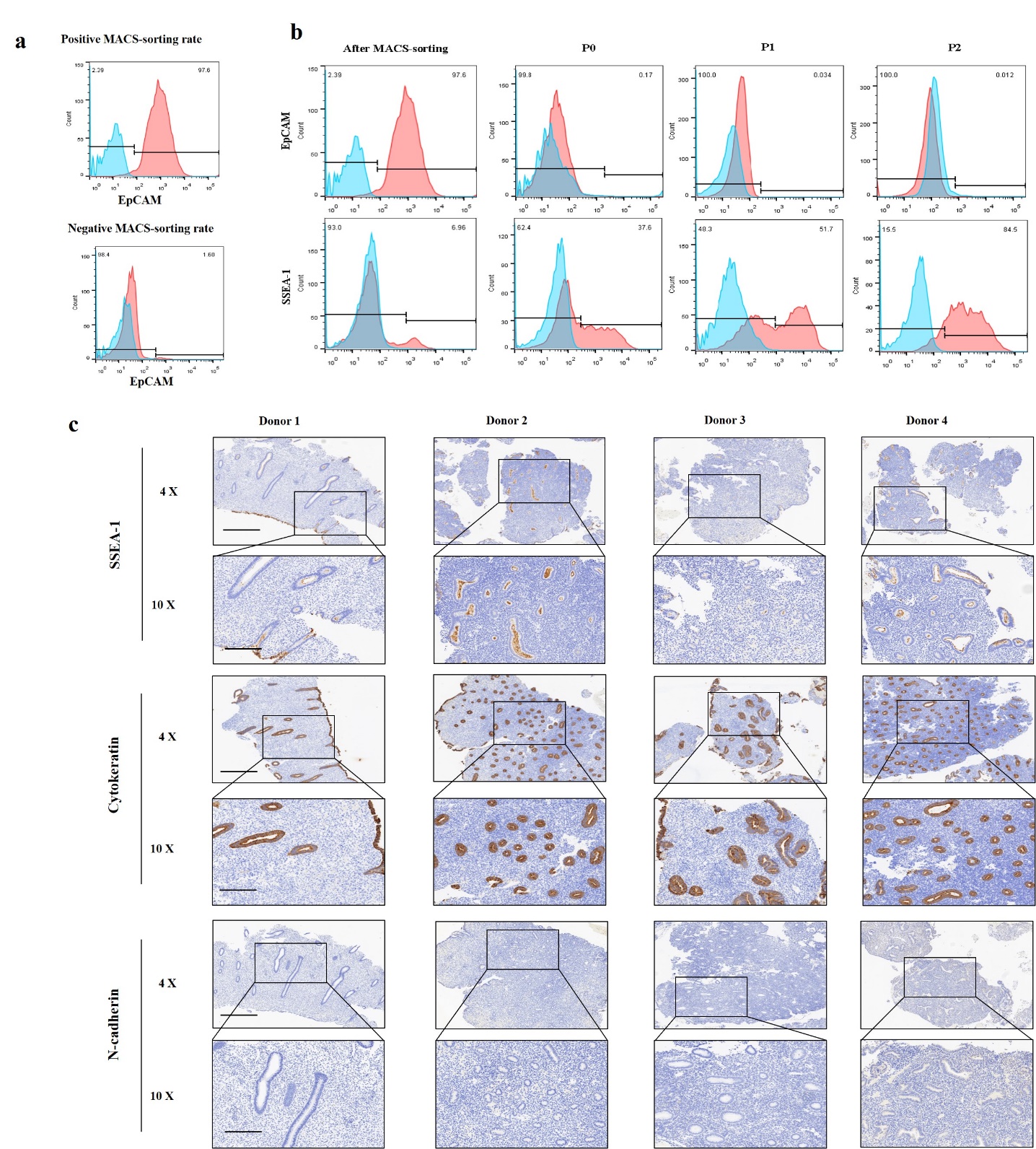


**FIGURE S1.** **Phenotypic characterization of human SSEA-1^+^ cells.** (**a**) Flow cytometric analysis showing the proportion of EpCAM^+^ cells after positive and negative MACS-sorting. (**b**) Flow cytometric analysis showing the tendency of EpCAM and SSEA-1 of EpCAM^+^ cells cultured in TEM. Red, positive-cells; blue, negative controls. (**c**) The SSEA-1, Cytokeratin and N-cadherin immunoexpression in the endometrium of proliferative phase. Scale bars, 500 µm in 4X, 200 µm in 10X.

**
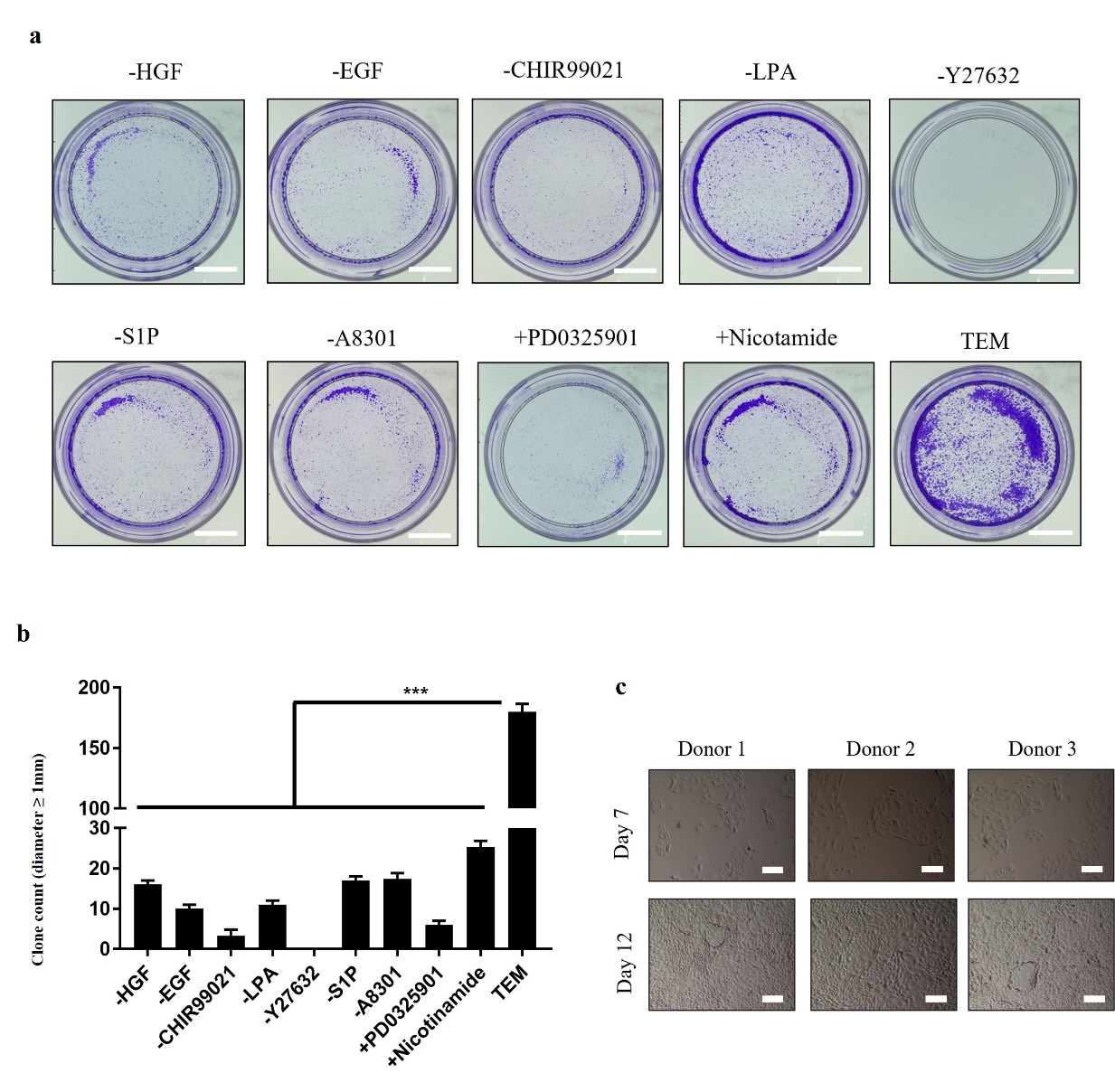
**

**FIGURE S2.** **Isolation and culture of human SSEA-1^+^ cells, related to Fig.1.** (**a**) Crystal violet staining of clones in TEM or TEN without HGF, EGF, CHIR99021, LPA, Y27632, S1P, A8301, or in TEM with PD0325901 or Nicotamide, respectively. Scale bars, 1 cm. (**b**) The clone numbers in TEM with or without factors. Error bars represent s.d.; n = 3 donors (*** *p*<0.001). (**c**) Light microscopy images of SSEA-1^+^ cells derived from 3 donors in TEM at passage 3. Scale bars, 100 µm.


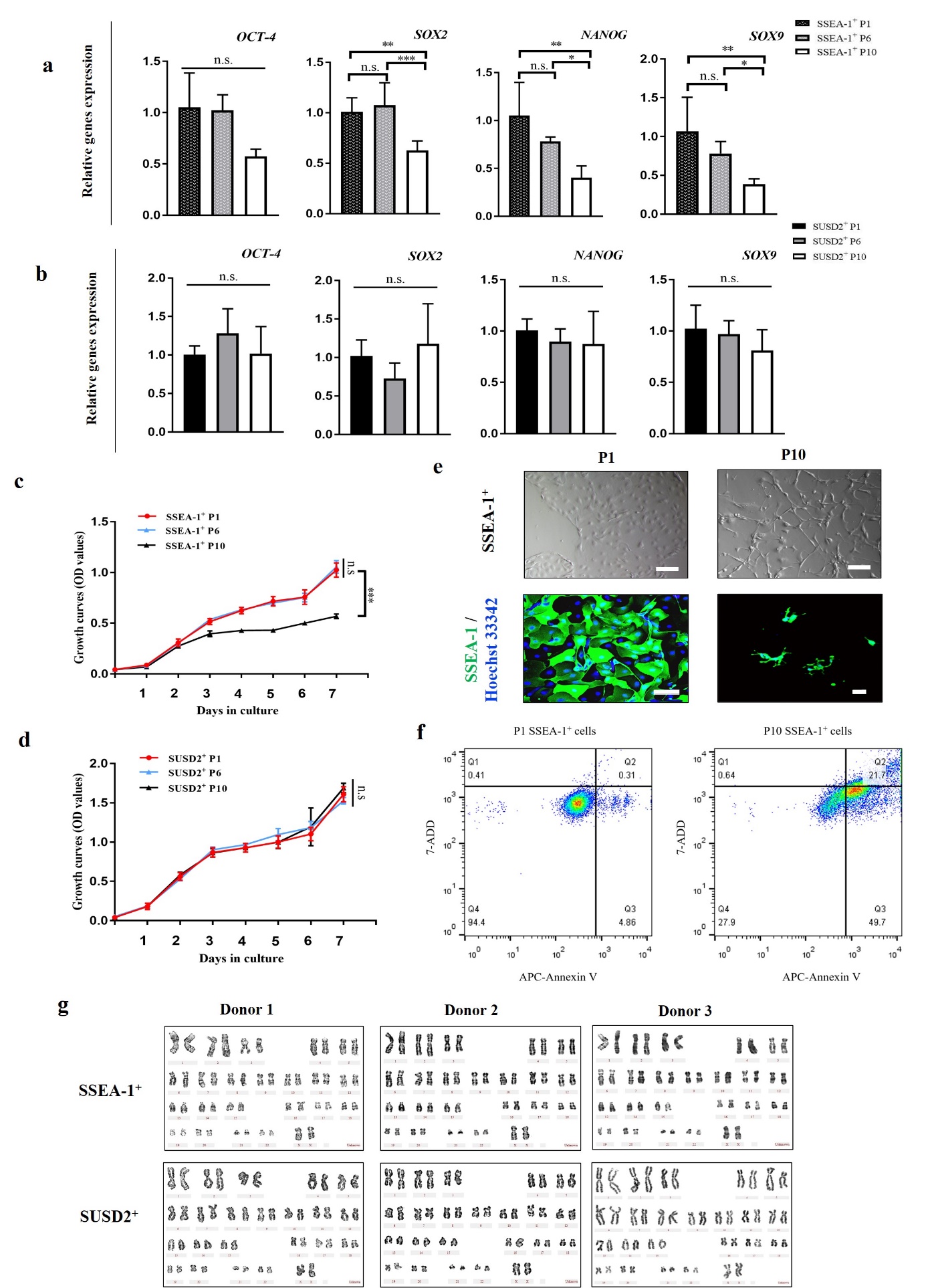


**FIGURE S3.** **Individual variations in proliferative potential of human SSEA-1^+^ and SUSD2^+^ cells from different donors.** (**a,b**) qPCR analyses for the expression of *OCT-4*, *SOX2*, *NANOG* and *SOX9* in SSEA-1^+^ cells (**a**) and SUSD2^+^ cells (**b**) at passage 1, passage 6 and passage 10. Expression normalized to *β-actin* (Error bars represent s.d., n = 9; n.s., non-significant; **P* < 0.05, ***P* < 0.01, ****P* < 0.001). (**c,d**) CCK-8 analyses of SSEA-1^+^ cells (**c**) and SUSD2^+^ cells (**d**) at passage 1, passage 6 and passage 10. (Error bars represent s.d., n = 3; n.s., non-significant; ****P* < 0.001). (**e**) Typical light microscopy images and immunofluorescence of SSEA-1^+^ cells at passage 1 and passage 10. Scale bars, 100 µm. (**f**) Cell apoptotic rates detected by flow cytometry for P1 and P10 SSEA-1^+^ cells. (**g**) Representative karyotype images of SSEA-1^+^ and SUSD2^+^ cells at passage 10.


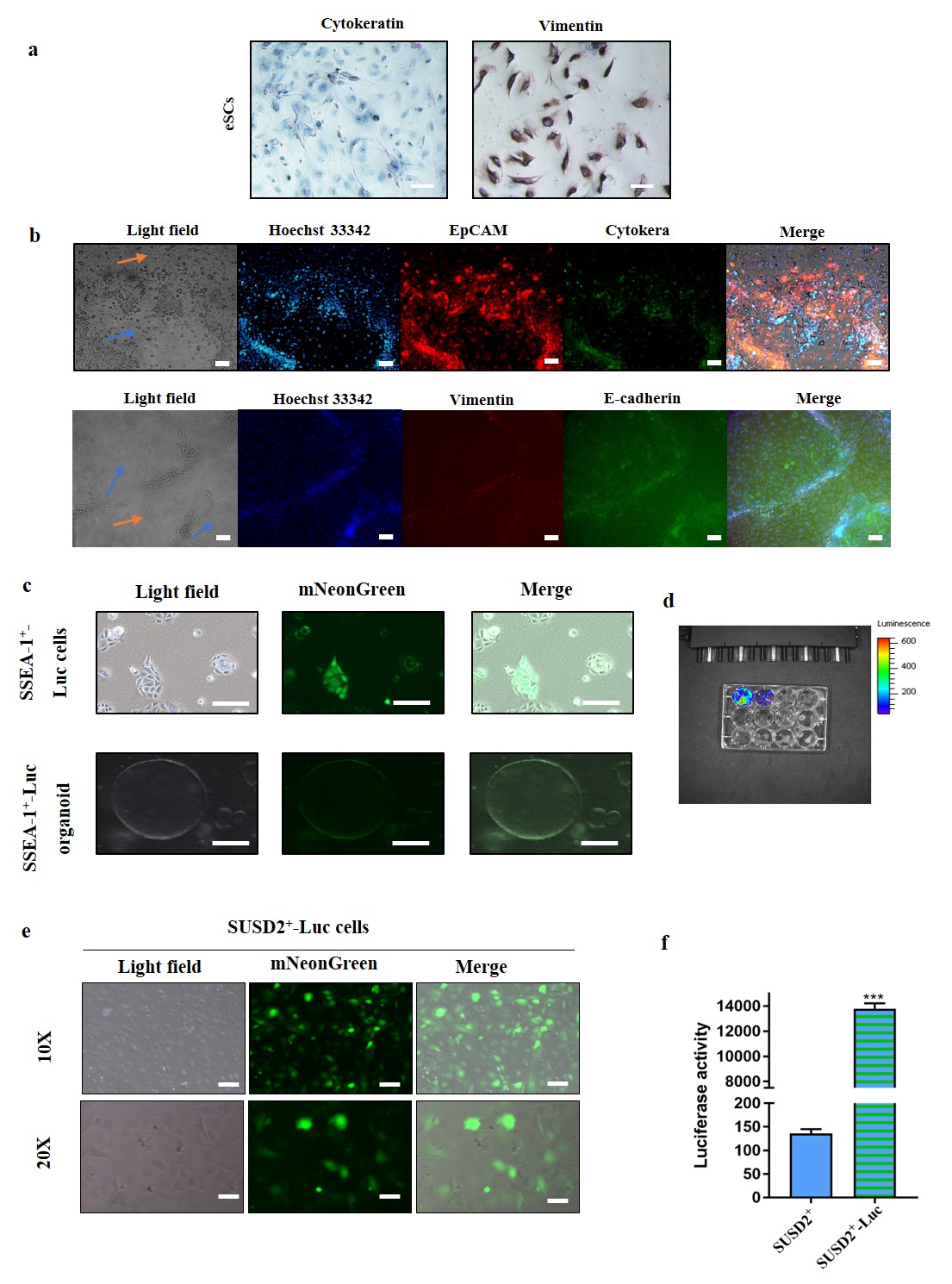


**FIGURE S4.** **The identification of eSCs, P0 endometrial cells, SSEA-1^+^ and SUSD2^+^ cells transduced with lentiviral vector expressing mNeonGreen and luciferase.** (**a**) Immunohistochemical identification of human endometrial stromal cells. Scale bars, 100 µm. (**b**) The light microscopy images and immunofluorescence analyses of P0 endometrial cells in TEM. Blue arrow, EpCAM^+^ cells; red arrow, EpCAM^-^ cells. Scale bars, 100 µm. (**c**) Representative image of SSEA-1^+^ cells and SSEA-1^+^ cells forming organoids transduced with lentivirus. Scale bars, 100 µm. (**d**) Fluorescent imaging of SSEA-1^+^ cells transduced with lentivirus in 12-well plate. (**e**) Representative image of SUSD2^+^ cells transduced with lentivirus. Scale bars, 100 µm. (**f**) The luciferase activity of SUSD2^+^ cells and SUSD2^+^ cells transduced with lentivirus detected by D-Luciferin sodium salt (Error bars represent s.d.; n = 3, two-tailed unpaired t-test, ****P* < 0.001).


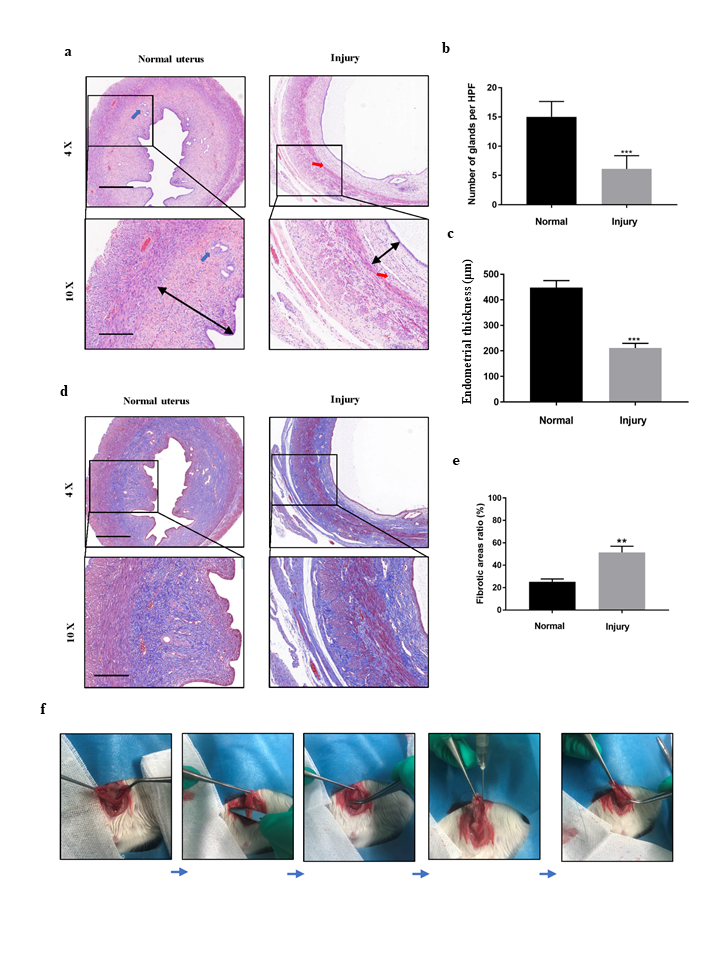


**FIGURE S5. The uterine horns at day 14 after injury.** (**a**) HE staining of uteri after different treatments for 14 days. Blue arrow, intact endometrial glands; red arrow, incomplete endometrial gland; black double-headed arrow, the endometrium thickness. Scale bars, 500 µm in 4X, 200 µm in 10X. (**b,c**) Statistical analysis of the number of glands (**b**) and the endometrium thickness (**c**) after injury (Error bars represent s.d.; n = 9; two-tailed unpaired t-test, ***P* < 0.01). (**d**) Collagen staining of uteri using Masson trichrome at day 14 after injury. Scale bars, 500 µm in 4X, 200 µm in 10X. (**e**) Statistical analysis of the percentages of collagen positive staining after injury (Error bars represent s.d.; n = 9; two-tailed unpaired t-test, ***P* < 0.01). (**f**) Schematic illustration of experimental procedures.


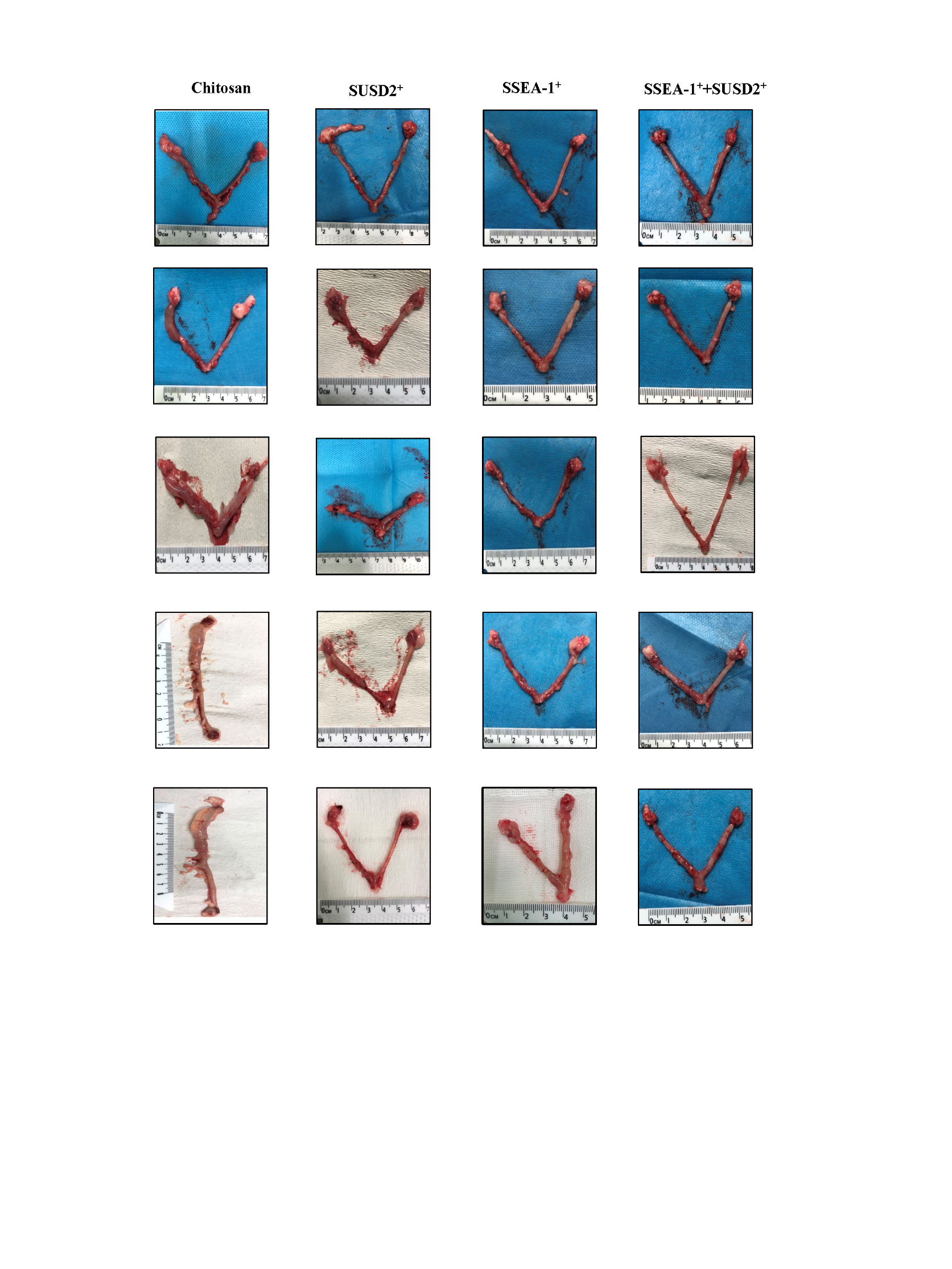


**FIGURE S6.** **Morphology of uteri in four groups following different treatments for 14 days.**

**SUPPLEMENTAL TABLES**

**Table S1. Primers used for q-PCR.**

| Primer name | Sequence (5′ → 3′) |
| --- | --- |
| human |  |
| TNF-F | CCCTGGTATGAGCCCATCTATC |
| TNF-R | AAAGTAGACCTGCCCAGACTCG |
| VEGFA-F | CCTTGCTGCTCTACCTCCAC |
| VEGFA-R | CACACAGGATGGCTTGAAGA |
| SOX9-F | GTACCCGCACTTGCACAAC |
| SOX9-R | TCTCGCTCTCGTTCAGAAGTC |
| SOX2-F | CTCCGGGACATGATCAGC |
| SOX2-R | CTGGGACATGTGAAGTCTGC |
| OCT-4-F | ACCGAGTGAGAGGCAACC |
| OCT-4-R | TGAGAAAGGAGACCCAGCAG |
| NANOG-F | CAACCAGACCCAGAACATCC |
| NANOG-R | TTCCAAAGCAGCCTCCAAG |
| IL1A-F | TGAAGGAGATGCCTGAGATACC |
| IL1A-R | GCAATAAACAAGTTTGGATGGG |
| IL1B-F | CATATGGCAGCTGTACGATCACTGAAC |
| IL1B-R | GCATCCTTAGGAAGACACAAATTGCAT |
| ITGB2-F | TGCGTCCTCTCTCAGGAGTG |
| ITGB2-R | GGTCCATGATGTCGTCAGCC |
| ITGB7-F | AGCAGCAACAACTCAACTGG |
| ITGB7-R | TTACAGACCCACCCTTCCTCT |
| NOTCH1-F | TTATGTAGTTGTTCGTTGGTTA |
| NOTCH1-R | AAAGGGTAGGATGCCTCCGTGT |
| CD9-F | GACACCTACAACAAGCTGAA |
| CD9-R | ACAGGACTTCACGGTGAAGG |
| CD13-F | GTAATACGACTCACTATAGGGCCAGGGGCCTGTACGTTTTTA |
| CD13-R | AATTAACCCTCACTAAAGGGCCACCAGCTCAGTCTTGTCA |
| EpCAM-F | CTGGTGTGTGAACACTGCTGGGG |
| EpCAM-R | TCTCCTTCTGAAGTGCAGTCCGC |
| E-cadherin-F | GAACGCATTGCCACATACAC |
| E-cadherin-R | AGCACCTTCCATGACAGACC |
| ER-F | TGATTGGTCTCGTCTGGCG |
| ER-R | CATGCCCTCTACACATTTTCCC |
| β-actin-F | AGACTTCGAGCAGGAGATGG |
| β-actin-R | CGGATGTCAACGTCACACTT |

**Table S2 Antibody list.**

| Antibody | Company | Ig Species | Dilution | Conjugate |
| --- | --- | --- | --- | --- |
| EpCAM | BioLegend | Mouse IgG | 1:20 (FC) | PerCP/Cy5.5 |
| Isotype control | BioLegend | Mouse IgG | 1:20 (FC) | PerCP/Cy5.5 |
| SUSD2 | BioLegend | Mouse IgG | 1:20 (FC) | PE |
| Isotype control | BioLegend | Mouse IgG | 1:20 (FC) | PE |
| CD34 | BioLegend | Mouse IgG | 1:20 (FC) | PE/Cy7 |
| Isotype | BioLegend | Mouse IgG | 1:20 (FC) | PE/Cy7 |
| SSEA-1 | BioLegend | Mouse IgM | 1:20 (FC) | APC |
| Isotype | BioLegend | Mouse IgM | 1:20 (FC) | APC |
| CD13 | BD Biosciences | Mouse IgG | 1:20 (FC) | BB515 |
| Isotype | BD Biosciences | Mouse IgG | 1:20 (FC) | BB515 |
| CD9 | BD Biosciences | Mouse IgG | 1:20 (FC) | BV421 |
| Isotype | BD Biosciences | Mouse IgG | 1:20 (FC) | BV421 |
| CD45 | BD Biosciences | Mouse IgG | 1:20 (FC) | APC-R700 |
| Isotype | BD Biosciences | Mouse IgG | 1:20 (FC) | APC-R700 |
| CD105 | BD Biosciences | Mouse IgG | 1:20 (FC) | BV421 |
| Isotype | BD Biosciences | Mouse IgG | 1:20 (FC) | BV421 |
| CD90 | BD Biosciences | Mouse IgG | 1:20 (FC) | BV510 |
| Isotype | BD Biosciences | Mouse IgG | 1:20 (FC) | BV510 |
| CD31 | BD Biosciences | Mouse IgG | 1:20 (FC) | APC-Cy7 |
| N-cadherin | BD Biosciences | Mouse IgG | 1:20 (FC) | PE |
| CD24 | BD Biosciences | Mouse IgG | 1:20 (FC) | FITC |
| SSEA-1 | Abcam | Mouse IgM | 1:200 (IHC) |  |
| SUSD2 | Abcam | Rabbit IgG | 1:150 (IHC) |  |
| Cytokeratin | Abcam | Mouse IgG | 1:250 (IHC) |  |
| Vimentin | Abcam | Mouse IgG | 1:250 (IHC) |  |
| N-cadherin | Abcam | Rabbit IgG | 1:250 (IHC) |  |
| Estrogen Receptor | Abcam | Rabbit IgG | 1:250 (IHC) |  |
| Donkey anti-Mouse IgG, Alexa Fluor 555 | Invitrogen |  | 1:500 |  |
| Goat anti-Mouse IgG, Alexa Fluor 488 | Invitrogen |  | 1:500 |  |
| Donkey anti-Rabbit IgG, Alexa Fluor 555 | Invitrogen |  | 1:500 |  |
| Goat anti-Rabbit IgG, HRP | Invitrogen |  | 1:5000 |  |
| rat-β-actin-R |  |  |  |  |

**Table S3 Donor information.**

| Identifier | Age | Menstrual cycle stage | Method of biopsy | Associated pathology |
| --- | --- | --- | --- | --- |
| Donor 1 | 38 | Day 12 | Curettage of uterus | proliferative endometrium |
| Donor 2 | 37 | Day 12 | Curettage of uterus | proliferative endometrium |
| Donor 3 | 32 | Day 11 | Curettage of uterus | proliferative endometrium |
| Donor 4 | 35 | Day 11 | Curettage of uterus | proliferative endometrium |
| Donor 5 | 31 | Day 12 | Curettage of uterus | proliferative endometrium |
| Donor 6 | 41 | Day 12 | Curettage of uterus | proliferative endometrium |
